# Psychological distance intervention reminders reduce alcohol consumption frequency in daily life

**DOI:** 10.1038/s41598-023-38478-y

**Published:** 2023-07-25

**Authors:** Mia Jovanova, Danielle Cosme, Bruce Doré, Yoona Kang, Ovidia Stanoi, Nicole Cooper, Chelsea Helion, Silicia Lomax, Amanda L. McGowan, Zachary M. Boyd, Dani S. Bassett, Peter J. Mucha, Kevin N. Ochsner, David M. Lydon-Staley, Emily B. Falk

**Affiliations:** 1 Annenberg School for Communication, University of Pennsylvania, Philadelphia, USA; 2 Desautels Faculty of Management, McGill University, Montreal, Canada; 3 Department of Psychology, Columbia University, New York, USA; 4 Department of Psychology, Temple University, Philadelphia, USA; 5 Mathematics Department, Brigham Young University, Provo, USA; 6 Department of Bioengineering, University of Pennsylvania, Philadelphia, USA; 7 Department of Electrical & Systems Engineering, University of Pennsylvania, Philadelphia, USA; 8 Department of Neurology, University of Pennsylvania, Philadelphia, USA; 9 Department of Psychiatry, University of Pennsylvania, Philadelphia, USA; 10 Department of Physics and Astronomy, University of Pennsylvania, Philadelphia, USA; 11 The Santa Fe Institute, Santa Fe, USA; 12 Department of Mathematics, Dartmouth College, Hanover, USA; 13 Leonard Davis Institute of Health Economics, University of Pennsylvania, Philadelphia, USA; 14 Department of Psychology, University of Pennsylvania, Philadelphia, USA; 15 Wharton Marketing Department, University of Pennsylvania, Philadelphia, USA; 16 Wharton Operations, Information and Decisions Department, University of Pennsylvania, Philadelphia, USA

**Keywords:** Psychology, Human behaviour

## Abstract

Modifying behaviors, such as alcohol consumption, is difficult. Creating psychological distance between unhealthy triggers and one’s present experience can encourage change. Using two multisite, randomized experiments, we examine whether theory-driven strategies to create psychological distance—mindfulness and perspective-taking—can change drinking behaviors among young adults without alcohol dependence via a 28-day smartphone intervention (Study 1, *N* = 108 participants, 5492 observations; Study 2, *N* = 218 participants, 9994 observations). Study 2 presents a close replication with a fully remote delivery during the COVID-19 pandemic. During weeks when they received twice-a-day intervention reminders, individuals in the distancing interventions reported drinking less frequently than on control weeks—directionally in Study 1, and significantly in Study 2. Intervention reminders reduced drinking frequency but did not impact amount. We find that smartphone-based mindfulness and perspective-taking interventions, aimed to create psychological distance, can change behavior. This approach requires repeated reminders, which can be delivered via smartphones.

## Introduction

Behaviors like alcohol use, smoking, and unhealthy eating are leading contributors to preventable disease and morbidity^1^. Creating psychological distance between unhealthy triggers and a person’s present experience—temporally, spatially, or socially,—may be an effective way to change behavior^2^. For example, creating ‘space’ from alcohol^3^, cigarette cues^4^, and unhealthy foods^5^ motivates healthier short-term choices in laboratory settings. Yet in everyday life, unhealthy triggers are abundant^6,7^.What tools can help people to create distance from unhealthy triggers and pursue healthier options as they go about their lives? A growing body of research highlights the promise of smartphone-delivered health reminders^8^. Since smartphones are ubiquitous and often with people as they go about daily lives, they offer important opportunities to test how to integrate theory-driven strategies from the laboratory into real-world settings^9^. Extending prior work, we focused on two popular psychological distancing strategies—mindfulness^10^ and perspective-taking^11^ to test the feasibility of smartphone reminders to change alcohol use behaviors among young adults. We deployed two multisite, randomized experiments: one prior to and another during the COVID-19 pandemic. The first experiment provided a proof concept; the second tested for scalability with a larger sample and without an in-person intervention component.

Alcohol use is a prevalent behavior^1^ that often holds an integral role in social interactions, particularly among young adults^12^. In the United States, alcohol use rates are some of the highest during the early twenties^13^ and drinking is disproportionately pervasive on college campuses where 80% of students report consuming alcohol^14^ as opposed to 64.8% in the general public^15^. Although alcohol often forms a common part of the college experience, with expectations that social drinking will lead to positive social experiences^16^ and bonding^17^, drinking in excess may also be detrimental^18^. Alcohol may increase behaviors that put young adults at risk for infectious and chronic diseases, as well as their communities through productivity losses, anti-social behavior, violence, and accidents^1^. Unhealthy drinking habits often develop in college^12^ and pose risks for future alcohol dependence and associated health problems^19^. Accordingly, we sought to identify preventative strategies that aim to counter, or divert, drinking behaviors among college students. In this vein, we developed two interventions that draw on psychological strategies—mindfulness and perspective-taking—as two proof of concept ways to create distance from alcohol cues and promote behavior change.

The first popular strategy, mindfulness, involves creating space between a stimulus (e.g., an alcoholic drink) and a person’s natural reaction to it^20^. Common mindfulness paradigms train individuals to take a step back and to accept their thoughts and feelings towards a trigger without judgment or avoidance. Theoretically, taking a step back may re-orient attention away from reactive thought patterns and dissuade unhealthy choices, possibly by de-automatizing responses to triggers^10,21^. Meta-analyses suggest that mindfulness training can be a promising component of substance use interventions^22,23^ across alcohol^24^, tobacco^25^, and cannabis and opiate use^26,27^, particularly within laboratory contexts and among clinical populations^28,29^. However, less work has leveraged mindfulness-based strategies as preventative efforts within more ecologically valid contexts, such as throughout daily life, and among non-clinical populations, such as healthy young adults. Among at-risk young adults, mindfulness interventions have produced mixed effects at reducing drinking^30–35^. Some studies suggest that a brief, in-person mindfulness training can decrease short-term consumption^36,37^. Others call for more frequent and higher intervention doses outside the laboratory^30,31^. Although distancing-based mindfulness strategies may be a promising path to behavior change, more research is needed on how to effectively incorporate these strategies into the daily lives of young adults, to possibly harness their preventative benefits^38^.

A second strategy to create psychological distance involves perspective-taking^11^. Grounded in social learning theory^39^, perspective-taking paradigms typically instruct individuals to imagine how a target person may think, feel, or behave in a given situation^40^. Imagining how a target would react, for example in response to an alcohol cue, can create distance by re-orienting attention away from a person’s immediate reaction to alcohol and towards the desired target’s response. Separate bodies of work support this view. First, neuroimaging evidence suggests that adopting another target’s perspective can shift neural responses to parallel the target’s affective experience^41^. Second, behavioral work suggests that making salient the health behaviors of relevant targets, such as those of health conscious peers, can encourage the adoption of healthy behaviors^42^, possibly by calling into mind the subjective value of peers’ health habits^43^ or by increasing perceived self-other overlap^44^. These separate literatures motivate a test of whether approaching alcohol cues from the perspective of a low-drinking peer target, with the goal to create distance from alcohol cues, can change drinking behaviors. In line with this possibility, correlational research suggests that individuals who score higher (vs. lower) on perspective-taking report lower susceptibility to peer drinking influences^45^ and lower drinking^46^. Importantly, this research is cross-sectional and did not consider real peers as perspective-taking targets, nor their drinking behavior. Extending prior work, we suggest that experimentally prompting individuals to take the perspective of peers whom they perceive to drink less than themselves may offer an opportunity to create psychological distance from alcohol cues and help change drinking behaviors in day-to-day life.

Overall, the evidence reviewed above suggests that mindfulness and perspective-taking strategies may reduce drinking by promoting different types of psychological distance. However, less is known about how to effectively integrate these strategies into individuals’ natural environments where pro-drinking influences may be present^6,7^. Indeed, current data is mixed, with some studies showing that brief psychological distancing trainings can motivate healthier choices^36,37^, and others suggesting that such trainings require boosts from ongoing reminders^30,31^. To address the possibility that repeated exposure is critical for the effectiveness of health communications in daily life^47^, we conducted randomized experiments using ecological momentary assessment (EMA), which collects intensive repeated measures in everyday settings^48^. We developed mindfulness and perspective-taking instructions designed to create psychological distance from alcohol cues and integrated them in the everyday lives of college students via smartphones. This approach allowed us to embed an experimental intervention while preserving a degree of ecological validity.

We randomly assigned participants to undergo a brief, in-person mindfulness, perspective-taking, or control training on how to respond to alcohol cues in their everyday life. Next, they received two smartphone reminders according to their respective condition and reported their alcohol use twice daily over the course of 28 days. We manipulated whether participants received psychological distance smartphone reminders (either mindfulness or perspective-taking) vs. control reminders, within-person over four alternating weeks, and examined changes in drinking frequency—the likelihood of having a drinking occasion, and drinking amount—the number of drinks per occasion^49^. Consistent with prior work, we modeled the frequency of drinking occasions and the amount consumed per occasion, as two separate outcomes, as both are independently linked to drinking habits and related health outcomes^50^. Our study focused on effects of psychological distancing reminders in general, and we had no a priori hypothesis about the relative effectiveness of the mindfulness versus perspective-taking strategies. We hypothesized that the frequency of alcohol occasions and the number of drinks per occasion would decrease on weeks when individuals received active intervention reminders versus control reminders. Further, we sought to replicate the effects of psychological distancing reminders on alcohol use frequency and amount with a larger sample and through a more scalable approach. Therefore, we conducted a second experiment that used fully remote delivery, which we refer to as Study 2. Study 1 provided a proof of concept during a regular time of in-person instruction when college students were on campus, prior to the COVID-19 pandemic. In turn, Study 2 presented a close replication study, conducted during the onset of the COVID-19 pandemic and throughout 2020.

## Results

### Intervention compliance, alcohol consumption, and baseline differences

In Study 1, participants were highly compliant with the protocol responding to a median of 94.64% of 56 EMA alcohol surveys sent to each person over the 28 days (*M* = 91.02%, *SD* = 12.72). In total, we collected 5492 data points on alcohol use. To measure the likelihood of a drinking occasion per assessment, participants responded to: “Since your EVENING/ MORNING survey, have you consumed any alcohol? (“No” or “Yes”). Participants who responded “Yes”, were asked to enter the number of standard servings of beer, liquor, and wine consumed:“Since the evening survey, how many standard servings (12 fl oz) of BEER/ (5 fl oz) of WINE /(1.5 fl oz) of LIQUOR have you had?”, using a numeric entry. To capture the total number of drinks per occasion, we summed responses for each beverage per assessment. See Refs.^51,52^ for previous use of these scales to capture alcohol use during daily life and the Measures section of the Methods for more details. These measures were also adapted from Ref.^53^ for twice-a-day morning and evening in situ assessment; with the original items previously validated against transdermal monitoring^53^. In Study 1, the average participant reported having an alcohol use occasion on 15.36% of all assessments and having 3.12 (*SD* = 1.70) drinks per occasion. Six participants (~ 6%) reported no alcohol use throughout the intervention period.

In Study 2, we made use of the same alcohol use measures as in Study 1. In Study 2, participants were highly compliant with the protocol responding to a median of 91.07% of 56 EMA alcohol surveys sent (*M* = 82.13, *SD* = 24.80). In total, we collected 9994 data points on alcohol use. The average participant reported having alcohol on approximately 10.12% of all assessments and having approximately 2.00 (*SD* = 1.13) drinks per occasion. Seventy-eight participants (~ 35%) reported no alcohol throughout the intervention period, which mainly took place after students left campus during the COVID-19 pandemic. See Table 1 and Table S1 for descriptive statistics by intervention week and condition.

**Table 1:** Descriptive Statistics.

Study 1										
Conditions	Mindfulness				Perspective-taking				Control	
Intervention weeks	Active weeks		Inactive weeks		Active weeks		Inactive weeks		N/A	
	*M*	*SD*	*M*	*SD*	*M*	*SD*	*M*	*SD*	*M*	*SD*
Percentage of EMA	89.96%	13.49%	91.22%	13.61%	86.44%	15.15%	92.54%	13.99%	92.86%	13.32%
responses										
Percentage of	12.63%	9.24%	15.59%	12.88%	12.94%	13.14%	15.53%	15.80%	17.61%	12.39%
drinking occasions										
Drinks per occasion	2.81	1.81	2.87	1.65	2.92	1.72	2.98	1.53	3.59	1.86

Study 2										
Conditions	Mindfulness				Perspective-taking				Control	
Intervention weeks	Active weeks		Inactive weeks		Active weeks		Inactive weeks		N/A	
	*M*	*SD*	*M*	*SD*	*M*	*SD*	*M*	*SD*	*M*	*SD*
Percentage of EMA responses	81.48%	22.39%	85.33%	24.03%	78.57%	30.55%	82.78%	29.02%	82.27%	23%
Percentage of	9.14%	15.02%	12.08%	15.54%	9.33%	14.80%	10.77%	15.00%	9.88%	12.07%
drinking occasions										
Drinks per occasion	2.39	1.81	2.46	1.42	1.93	0.97	1.71	1.16	1.83	1.05

*M* and *SD* represent mean and standard deviation, respectively.

**Figure 1:**
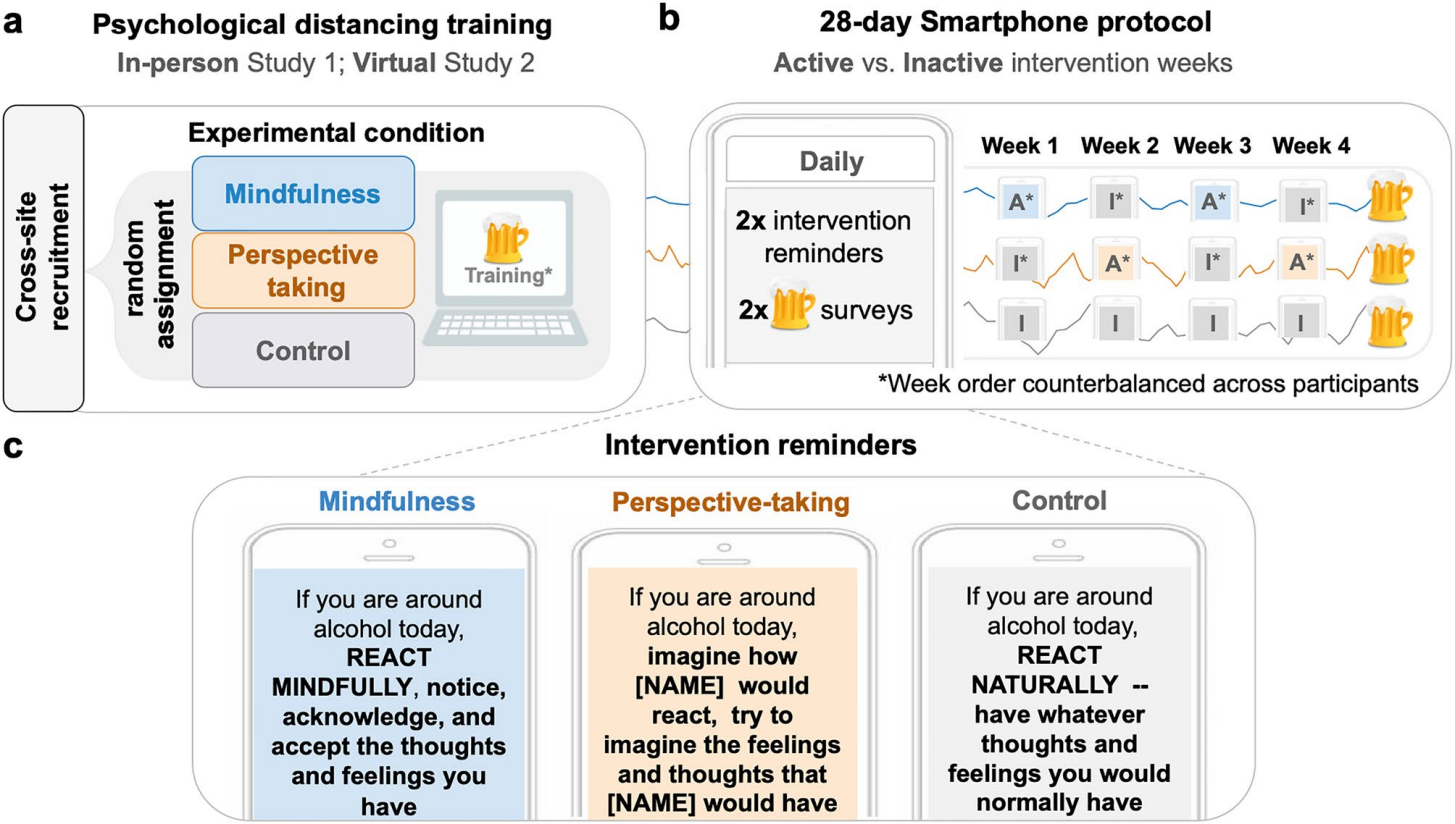
Study procedure and intervention reminders. We conducted two randomized experiments which we refer to as Study 1 and Study 2. a. Young adults (Study 1: *N* = 108; Study 2: *N* = 218), recruited across two urban university sites in the Northeastern United States, completed online surveys and were randomly assigned to one of three experimental conditions to complete a psychological training (in-person Study 1; or virtual Study 2) on how to respond to alcohol cues. b. Following the training, participants underwent a 28-day smartphone intervention in which they received two intervention reminder texts a day and two texts assessing their alcohol use. Participants in the mindfulness and perspective-taking conditions, received active intervention reminders on one week and control reminders the following week, with week order counterbalanced across participants. c. Reminder text messages corresponding to condition assignment and week.

Next, we report group differences on key variables across both studies. Response rates during the intervention protocol did not vary by condition assignment: Study 1: *F*(2, 105) = 0.64, *p* = 0.526; Study 2: *F*(2, 215) = 0.22, *p* = 0.801. Participants in the mindfulness and perspective-taking conditions responded to more alcohol surveys on inactive vs. active weeks (Study 1: inactive weeks (*M* = 25.72; *SD* = 3.84) vs. active weeks (*M* = 24.72, *SD* = 4.00), *t*(70) = −2.99, *p* = 0.024; Study 2: inactive weeks *(M* = 23.54; *SD* = 7.42) vs. active weeks (*M* = 22.41, *SD* = 7.46), *t*(146) =—3.47, *p* =  < 0.001). To account for these differences in response rates on active versus inactive weeks, we controlled for the number of responses to survey prompts in analyses (see Data Analysis section of the Methods section). We observed no group differences in age, gender and race (Study 1 age: *F*(2, 105) = 0.98, *p* = 0.376; gender: *X*^2^(4, *N* = 108) = 2.75, *p* = 0.685; race: *X*^2^(8, *N* = 108) = 3.06, *p* = 0.9307); Study 2 age: *F*(2, 203) < 0.01, *p* = 1; gender: *X*^2^ (2, *N* = 206) = 0.98, *p* = 0.613; race: *X*^2^(8, *N* = 206) = 3.76, *p* = 0.878). With respect to differences in baseline drinking frequency, in Study 1, the perspective-taking group (*M* = 0.90; *SD* = 0.45) reported fewer alcohol occasions in the month prior to the intervention relative the control group (*M* = 1.06; *SD* = 0.56; β = −0.27; *p* = 0.016), highlighting a failure of randomization to create equal groups. We observed no other differences in baseline drinking frequency across other group comparisons (mindfulness vs. control: β = −0.15; *p* = 0.186; perspective-taking vs. mindfulness: β = −0.13; *p* = 0.264), and no group differences in the number of drinks in the month prior to the intervention (*F*(2, 98) = 0.508,* p* = 0.6033) in Study 1. See Table 2 for descriptive statistics of baseline alcohol use by group. We did not collect baseline drinking information for participants in Study 2 (Fig. 1).

**Table 2:** Participant characteristics by group.

Baseline characteristic	Study 1						Study 2					
	Mindfulness n = 37		Perspective- taking n = 37		Control n = 34		Mindfulness n = 75		Perspective- taking n = 72		Control n = 71	
	Mean or n	SD or %	Mean or n	SD or %	Mean or n	SD or %	Mean or n	SD or %	Mean or n	SD or %	Mean or n	SD or %
Age	20.70	(1.87)	20.21	(1.59)	20.70	(1.65)	20. 60	(1.26)	20.60	(1.50)	20.60	(3.16)
Missing Gender	–	–	–	–	–	–	3	4.0%	5	6.9%	4	5.6%
Women	21	56.8%	22	64.71%	22	59.46%	58	77.3%	49	68.1%	50	70.4%
Men	15	40.5%	12	35.29%	15	40.54%	14	18.7%	17	23.6%	17	23.9%
Other	1	2.7%	0	0%	0	0%	0	0%	0	0%	0	0%
Missing (n, %)	–	–	–	–	–	–	3	4.0%	6	8.3%	4	5.6%
Race												
white	20	54.1%	19	55.9%	19	51.40%	24	32.0%	29	40.3%	28	39.4%
Asian	13	32.4%	9	26.5%	11	29.7%	29	38.7%	19	26.4%	21	29.6%
Black	1	2.7%	1	2.9%	0	0%	6	8.0%	6	8.3%	5	7.0%
Latino/a	1	2.7%	1	2.9%	3	8.1%	5	6.7%	3	4.2%	3	4.2%
Other	3	8.1%	4	11.8%	4	10.8%	8	10.7%	10	13.9%	10	14.1%
Missing	–	–	–	–	–	–	3	4.0%	5	6.9%	4	5.6%
Baseline alcohol use												
Drinking frequency	0.90	(0.45)	0.76	(0.52)	1.06	(0.56)	–	–	–	–	–	–
Missing (n, %) Drinking amount Missing (n, %)	3 2.43 3	8.1% (1.71) 8.1%	2 2.80 2	5.4% (2.89) 5.4%	1 2.92 2	2.9% (1.50) 5.8%	–	–	–	–	–	–

Baseline drinking measures were only collected in Study 1, but not in Study 2. Participants reported their habitual beer, wine, and spirits consumption per week in the month prior to the intervention using the Alcohol Use Questionnaire (AUQ)^81^. To create the baseline drinking measures, we averaged the number of drinks and number of drinking occasions across beer, wine, and spirits.

### Within-person effects of daily reminders on alcohol consumption

**Figure 2:**
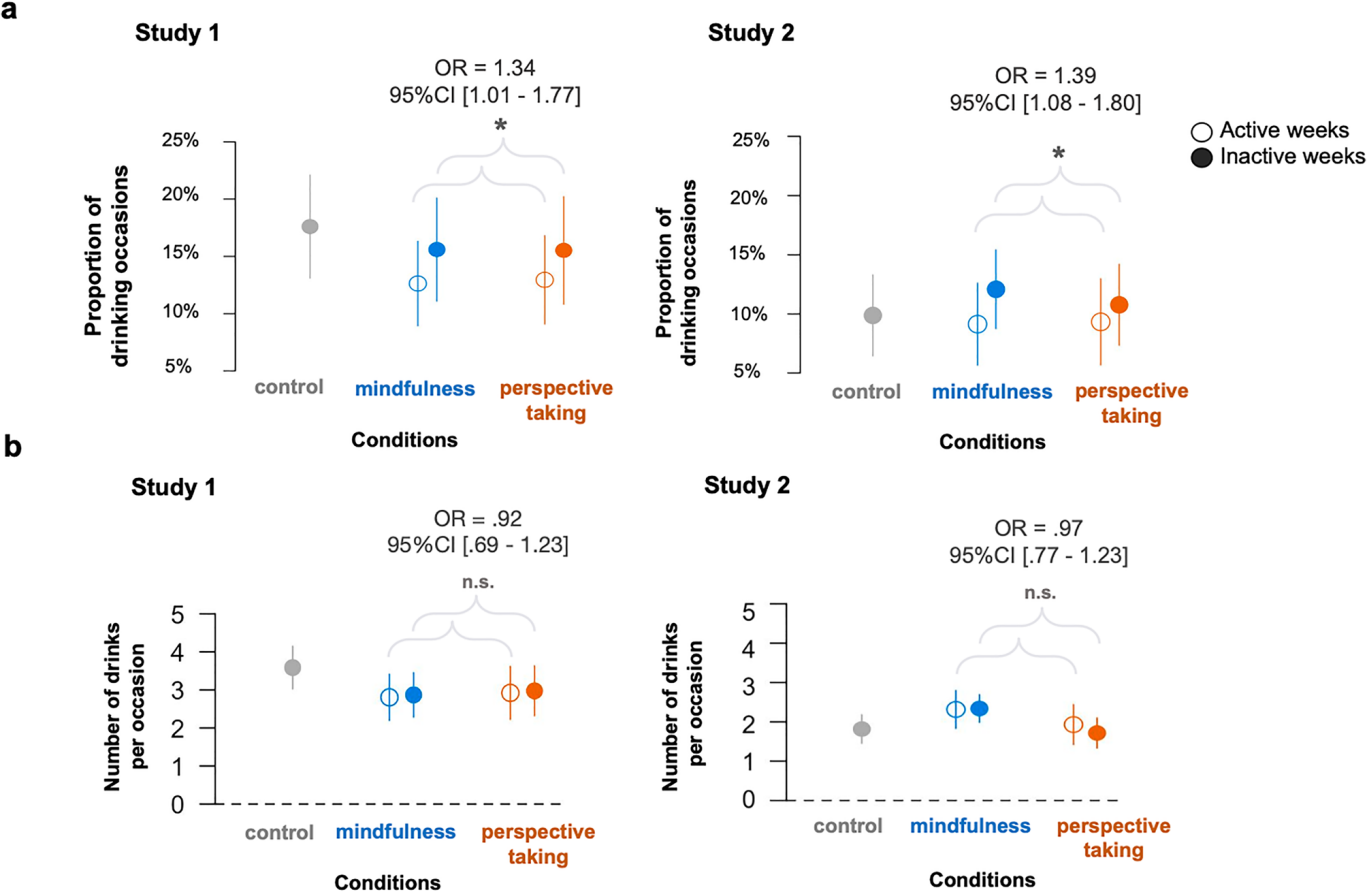
Psychological distance reminders reduce drinking frequency but do not influence amount. a. Collapsing across experimental conditions, participants in the mindfulness and perspective-taking groups were less likely to drink following active intervention reminders relative to following control reminders in Study 1 (left) and Study 2 (right). b. We found no differences in the number of drinks consumed on alcohol use occasions following active intervention reminders, relative to following control reminders across both studies, Study 1 (left) and Study 2 (right). Note: figure presents raw data for illustration. Dots present the mean and the error bars present 95% confidence intervals.

We tested the feasibility of psychological distancing reminders to reduce the number of drinking occasions and drinks per occasion on active, intervention weeks versus inactive, non-intervention weeks. To assess differences in drinking on active versus inactive weeks, we specified two separate multilevel hurdle models, one for Study 1 and another for Study 2. The main predictor was active week (vs. inactive week). Consistent with prior work^50^, we separately examined two outcomes: frequency of drinking occasions and number of drinks per occasion (see Data Analysis of the Methods section for information on covariates and modeling details). We report effects on drinking occasion frequency first and drinking amount second. The results are visualized in Fig. 2.

#### Psychological distance reminders reduce drinking frequency

Examining both distancing strategies together (i.e., collapsing across mindfulness and perspective-taking conditions), we found a directional main effect of the intervention reminders such that participants in the mindfulness and perspective-taking conditions were less likely to drink on active weeks (following active reminders) relative to inactive weeks (following control reminders) (active week vs. inactive week: OR = 1.34, 95% CI [1.01–1.77], *p* = 0.041; Table 3), though this effect was marginal after correction for multiple comparisons in Study 1 (FDR corrected* p* = 0.082). We found significant main effects in Study 2, such that participants in the mindfulness and perspective-taking conditions were significantly less likely to drink on active weeks (following active reminders) relative to inactive weeks (following control reminders)(active week vs. inactive week: OR = 1.39, 95% CI [1.08–1.80], *p* = 0.011, FDR corrected* p* = 0.022, Fig. 1; Table 3). These results were obtained from a zero-inflated hurdle model, to account for the fact that participants had zero drinks on some days. The zero-inflated sub-model estimated the probability of an extra zero (no alcohol use) such that a positive odds ratio indicates more occasions with no alcohol use. See Data Analysis section of the Methods section for modeling details. These results offer support that psychological distancing strategies, drawing on mindfulness and perspective-taking, may call for repeated reminders to reduce drinking frequency in our samples of non-alcohol dependent college students.

**Table 3:** Within-person effects of reminders on drinking on active vs. inactive weeks.

Fixed effects	Study 1			Study 2		
	Zero-inflated sub-model					
	OR	95%CI	*p*	OR	95%CI	*p*
Intercept	1.77	[.26–11.8]	.558	10.4	[2.96–36.5]	< .001
Active week (vs. inactive)	1.34	[1.01–1.77]	.041	1.39	[1.08–1.80]	.011
Perspective condition (vs. mindful)	1.21	[.71–2.05]	.489	1.06	[0.60–1.87]	.845
Signal count	1.01	[1.01–1.02]	< .001	1.00	[0.99–1.00]	.458
Number of responses	1.03	[0.99–1.07]	.149	1.02	[1.00–1.04]	.109
Social weekend (vs. week)	0.50	[0.40- .61]	< .001	0.54	[0.45–0.65]	< .001
Perspective condition (vs. mindful) *active week (vs. inactive)	0.93	[0.62- 1.41]	.741	0.95	[0.66–1.38]	.796

Random effects	Variance	SD	Variance	SD		
Intercept						
Participant ID* Group ID	< .011	.938	.200	.447		
Participant ID	< .001	< .001	.079	.282		

Fixed Effects	Conditional sub-model					
	OR	95%CI	*p*	OR	95%CI	*p*
Intercept	3.56	[0.93–13.7]	.065	1.79	[0.87–3.68]	.111
Active week (vs. inactive)	0.92	[0.69–1.23]	.575	0.97	[0.77–1.23]	.816
Perspective condition (vs. mindful)	1.02	[0.69–1.51]	.919	.054	[0.38–0.76]	< .001
Signal count	1.00	[0.99–1.00]	.158	1.01	[1.00–1.01]	.041
Number of responses	0.99	[0.96–1.01]	.284	0.99	[0.98–1.01]	.353
Social weekend	1.56	[1.26–1.93]	< .001	1.27	[1.06–1.53]	.009
Perspective condition (vs. mindful) *active week (vs. inactive)	1.09	[.72–1.65]	.687	1.27	[0.89–1.80]	.186

Random effects	Variance	SD	Variance	SD		
Intercept						
Participant ID* Group ID	229	.478	1.879	1.371		
Participant ID	.028	.167	.738	.859		

Study 1: 3577 observations nested within 71 participants across 10 groups; Study 2: 6729 observations nested within 147 participants across 23 groups. The zero-inflation sub-model of the hurdle model estimates the probability of an extra zero (no alcohol use) such that a positive estimate indicates a higher chance of no alcohol use. Perspective = perspective-taking; mindful = mindfulness.

#### Psychological distance reminders do not impact drinking amount

Examining both distancing strategies together (i.e. collapsing across the two intervention conditions), we found no difference in the number of drinks consumed on alcohol use occasions on active weeks (following active intervention reminders) versus on inactive weeks (following control reminders) across both studies (Study 1 active week vs. inactive week; OR = 0.92, 95% CI [0.69–1.23], *p* = 0.575, Study 2: active week vs. inactive week: OR = 0.97, 95% CI [0.77–1.23], *p* = 0.816, Table 3). These results come from the portion of the hurdle model that examined how much alcohol is consumed, conditioned on drinking at all. Together, these results suggest that the distancing reminders helped reduce the drinking occasion frequency but did not influence the amount consumed when drinking.

#### Exploratory interaction effects of mindfulness vs. perspective-taking reminders

We next explored whether the effects of the intervention reminders varied based on distancing strategy: mindfulness vs. perspective-taking. To examine this question, we added an interaction term between active (vs. inactive) week and distancing strategy in each multilevel hurdle model, for Study 1 and Study 2 separately. We found no significant interaction between active (vs. inactive) week and mindfulness (vs. perspective-taking) on drinking frequency (Study 1: active week vs. inactive week: OR = 0.93, 95% CI [0.62–1.41], *p* = 0.741; Study 2: active week vs. inactive week OR = 0.95, 95% CI [0.66–1.38], *p* = 0.796, Table 3). This finding, as well as inspection of both raw and winsorized means (Table S1), suggests that both mindfulness and perspective-taking strategies, with frequent reminders, may be similarly effective in reducing drinking frequency. However, exploratory inspection of the medians by week and intervention strategy suggests that the mindfulness-based distancing reminders may be more robust in reducing drinking frequency (see Table S1). Further, we found no significant interaction between intervention week and distancing strategy on drinking amount (Study 1: active week vs. inactive week: OR = 1.09, 95% CI [0.72–1.65], *p* = 0.687; Study 2: active week vs. inactive week: OR = 1.27, 95% CI [0.89 – 1.80], *p* = 0.189, Table 3).

### Differences in behavior change of daily reminders on drinking frequency

We conducted follow-up analyses to explore whether the within-person effects of the psychological distance intervention reminders, observed in both studies, i.e. the decrease in the drinking occasion frequency from inactive to active weeks, differed from non-intervention related changes in the absence of distancing reminders. Specifically, we compared whether changes in drinking frequency in the intervention conditions differed from changes in drinking frequency in the control condition across both samples. As part of the study protocol, the control condition received control reminders throughout the entire study period, which prompted participants to respond to alcohol naturally. Thus, we were interested in whether the observed change in drinking frequency differed from change we may expect for participants self-monitoring their own alcohol use and completing other aspects of the protocol, but without the key psychological distancing components. To test for this possibility, we randomly labeled two of the four weeks as pseudo “active” and pseudo “inactive” in the control condition, despite the protocol remaining the same throughout. This random assignment allowed us to match the control and intervention protocol as closely as possible. We repeated this randomization 100 times and compared the average pseudo-inactive-to-active-week change scores for the control group to the change scores for the intervention groups. Performing this comparison allowed us to test whether the changes in drinking frequency among participants in the intervention conditions differed from changes among participants who participated in all other aspects of the study but were not instructed to adopt psychological distance. See Follow-up behavior change analyses section of the Methods for details.

### Psychological distance reminders reduce drinking frequency vs. control

**Figure 3:**
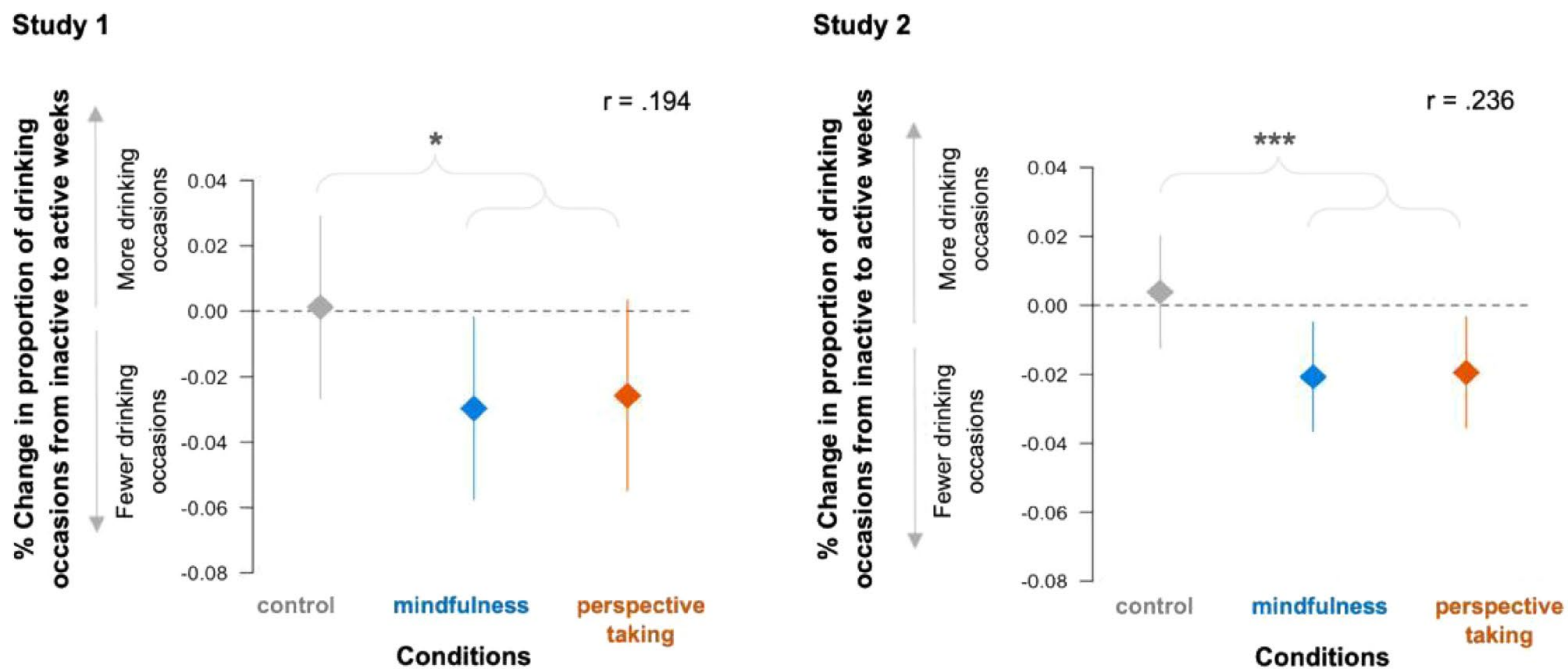
Intervention reminders change drinking frequency among participants in the intervention conditions vs. control. Participants in the distancing conditions—mindfulness and perspective-taking—reported greater behavior change, i.e., drank less frequently on active vs. inactive weeks, relative to the change among participants in the control condition who received non-intervention reminders, in Study 1 (left) and Study 2 (right). Negative change scores suggest intervention consistent behavior change, or decreases in the frequency of drinking occasions.

Examining both distancing conditions together (i.e., collapsing across mindfulness and perspective-taking conditions), we found that individuals in the intervention groups showed a greater decrease in drinking frequency from inactive weeks to active weeks relative to the control group, both in Study 1 (*W* = 1002, *Z* = −2.013, *p* = 0.044, *r* = 0.194) and in Study 2 (*W* = 3733, *Z* = −3.487, *p* =  < 0.001, *r* = 0.236; Fig. 3). We found parallel results when accounting for outliers (see Supplement C for more details). Together, these results further support the inference that the psychological distancing components reduced drinking frequency rather than extraneous factors unrelated to the psychological distancing interventions.

### Additional between-person group analyses

We explored overall group differences in drinking occasions and drinks per occasion and report these results in Supplementary Analyses D. We tested whether the intervention groups drank less frequently and had fewer drinks on active weeks, and across all weeks collapsed, relative to the control group. Briefly, we found no group differences in the frequency of drinking occasions (see Tables S3 and S5). The perspective-taking group reported fewer drinks per occasion relative to the control group in Study 1. However, we did not replicate these effects in Study 2 (see Tables S4 and S6). We are cautious to make strong claims about between-person effects given the possibility that random assignment did not fully overcome baseline differences between groups. In Study 1, we observed that the perspective-taking group reported drinking less frequently at baseline relative to the control group. Since we did not collect baseline drinking information for participants in Study 2, we cannot account for potential pre-existing differences between groups in Study 2 (see Table 2).

## Discussion

Changing behavior is difficult. Yet changing from unhealthy to healthy choices in day-to-day life, and preventing unhealthy choices, can improve long-term quality of life and longevity. Across two studies, we leveraged smartphones to administer two theory-driven interventions to change an important health behavior—alcohol use—among two samples of college students, as a basic science, proof of concept investigation. Within-person, we tested whether psychological distancing reminders, drawing on mindfulness and perspective-taking can decrease the frequency of alcohol occasions and the number of drinks per occasion on intervention weeks relative to non-intervention weeks over 28 days. We found that the psychological distance conditions drank directionally less frequently on active weeks, relative to inactive weeks in Study 1; and significantly less frequently on active weeks, relative to inactive weeks in Study 2, with a larger sample. Reminders, focused on creating psychological distance from alcohol cues, helped reduce the frequency of alcohol use occasions but did not impact the number of drinks consumed per occasion, within-person.

On average, across both studies, the frequency of drinking occasions decreased about 2%, on weeks when participants received twice-a-day psychological distancing reminders. In Study 1, drinking occasion frequency decreased from once every 6 days to once every 8 days when intervention reminders were present. In Study 2, drinking occasion frequency reduced from once every 9 days to once every 11 days. These effects are meaningful in light of broader goals to motivate positive behavior change in daily life from a preventative lens. Decreasing the frequency of alcohol occasions is not the direct goal of most alcohol harm reduction interventions among young adults, which often target binge drinking^54^. Our work provides proof of concept support for the feasibility of preventative, behavior change interventions that integrate psychological distancing strategies through smartphones, which are ubiquitous in day-to-day life. This basic science work encourages further development of theory-driven, smartphone interventions with cost-saving potential^55^ to prevent unhealthy choices in everyday settings and to preempt the development of more harmful behaviors (i.e., binge drinking)^54^.

Our investigation builds on bodies of research that consider psychological distancing strategies, such as mindfulness^22^, and the use of smartphones to promote behavior change^56^. Prior work has shown mixed effectiveness of smartphone interventions to change drinking behaviors, particularly among young adults^32–35,57^. Our two experiments extend the feasibility of theory-driven reminders that promote two kinds of psychological distance– mindfulness and perspective-taking– among college students without alcohol dependence. Consistent with prior work, our psychological distancing intervention effects on drinking frequency, may, to some degree, be contingent on repeated reminders. For example, Witkiewitz et al., found that randomizing college students into a 14-day smartphone-based intervention did not reduce drinking. However, among participants randomized to the smartphone-administered intervention, receiving more (vs. less) frequent personalized feedback modules, was associated with a lower likelihood of any drinking throughout the 14-day assessment^31^.

One possibility is that repeated psychological distance reminders help offload cognitive effort when evaluating health-related options^58^, particularly in key moments when individuals encounter alcohol cues in day-to-day-life. For example, distancing reminders may make healthier options more salient, by creating mental space from automatic reactions to triggers^59^, or by moving attention towards a healthier peer’s perspective. Another possibility, drawing on the alcohol cognitive bias modification literature^60,61^, is that distancing reminders may help de-automatize cognitive biases to alcohol cues encountered in daily life. For instance, social drinkers^62^ may allocate quicker attention to, and make more positive subjective evaluations^63^ of alcohol (vs. non-alcohol) cues. Cognitive debiasing tools, such as psychological distancing, may help counter attentional and subjective evaluation biases and downregulate reactivity when approaching alcohol cues^64^. Similar to our findings, research that leverages cognitive bias modification to shift attentional bias away from alcohol also suggests little success to reduce drinking over a single training session^65^, but more encouraging results over repeated retraining^66^. Further, distancing reminders may also work by helping individuals notice when there is a need to regulate and/or how they can go about doing so, more broadly^67^.

With respect to intervention specificity, both the mindfulness and perspective-taking reminders reduced drinking frequency on active vs. inactive weeks to a similar degree. It is possible that different types of psychological distancing strategies, with repeated reminders, may be similarly effective in reducing the frequency of drinking occasions relative to an individual’s own baseline. Further, it is plausible that both interventions leverage a parallel psychological distance mechanism, though our data do not speak to this possibility directly. For example, neuroimaging research suggests that different forms of psychological distance are encoded similarly in the brain^68,69^. However, future research that investigates these parallels in the drinking intervention context^70^ can help clarify the mechanisms through which psychological distancing strategies facilitate behavior change.

While promising, our intervention effects on reducing the frequency of drinking occasions should be interpreted with caution. We observed directional (Study 1) and significant (Study 2) within-person changes in drinking frequency from active to inactive weeks; with this change being greater than in the absence of active intervention reminders. However, participants in the intervention conditions did not necessarily drink less frequently than those assigned to the control condition, on average. One possibility is that random assignment did not completely overcome pre-existing differences in alcohol use between groups (see Intervention compliance, alcohol consumption and baseline differences in the Results section for more details). There also remains the possibility that the distancing reminders altered drinking by making individuals more reactive to being monitored, though participants also received instructions about how to approach alcohol and reported their drinking behavior daily during “inactive” weeks. Thus, our comparison likely represents a relatively conservative test of potential effects of distancing reminders on drinking behavior. Nevertheless, self-monitoring alcohol use may promote behavior change^34^. As such, future replication work may help isolate possible reactivity effects and build greater confidence in the distancing interventions.

Our results should also be interpreted considering design strengths and limitations. First, we employed theory-driven interventions within an EMA design, which allowed us to capture intervention effects in participants' natural environments and overcome common retrospective biases that ask participants to recall information about longer periods of time (e.g., amount of alcohol consumed in the previous 30 days). Next, the within-person manipulation allowed us to detect intervention effects while considering individuals’ personal drinking baselines. We employed this repeated measures design across two different samples, and across two different sites, thereby increasing the robustness of our findings. Further, we replicated these findings in a highly unusual and stressful time, the onset of the COVID-19 pandemic. Although intensive assessment often raises data compliance concerns, we observed little evidence of non-compliance in our data. The intensive sampling approach produced high response rates (approximately 95% median response rate in Study 1, with an in-person training component, and 91% median response rate in Study 2 with a fully remote delivery and a doubled sample size). These high response rates may support the feasibility of applying this approach for evaluating future interventions among college samples.

It is important to note that our data cannot speak to long-term intervention effects on drinking beyond the length of 28 days, and our results may not generalize to samples beyond college students in urban college campuses in the Northeastern United States without alcohol dependence who are part of social groups. Specifically, we recruited students from pre-existing social groups (and included groups in which 80% or more expressed interest), with higher proportions of women than men. Although we controlled for non-independence of observations in statistical modeling, the non-independence may have confounded intervention effects in unmeasurable ways (e.g., spillover effects or social influence). Finally, when interpreting results, it is important to note that the psychological distancing reminders did not explicitly instruct individuals to decrease the quantity of alcohol consumed. As such, future work could test more explicit instructions to directly target the number of drinks when drinking. Finally, we used self-reported measures of drinking which may be subject to social desirability bias and may underestimate drinking levels^71^. To reduce concerns over self-reported drinking, future research could incorporate measures of passive transdermal sensors^72^, or blood alcohol content reports via smartphones^73^.

## Conclusion

The present study responds to calls to develop more effective, theoretically-guided behavior change interventions^8,74^. The two mobile-health interventions tested here contribute to a growing literature on how to leverage mobile technology to administer psychological behavior change interventions among young adults. In two multi-site, randomized experiments we found that psychological distance-based reminders—drawing on mindfulness and perspective-taking—and delivered via smartphones, helped decrease the frequency of drinking occasions over the course of a month, both prior and during the onset of the COVID-19 pandemic. Intervention reminders specifically helped reduce drinking frequency but had little impact on the amount consumed per occasion. Future research may expand the use of psychological-distancing interventions employed via smartphones to motivate behavior change outside the lab.

## Methods

We use data from two different cohorts of college students from the Social Health Impact of Network Effects (SHINE) Study which we refer to as Study 1 and Study 2. Details of recruitment, study design, and data analysis can be found in Supplement A. All research, methods, and study protocols were approved by the Human Subjects Electronic Research Application (HSERA) Institutional Review Board (IRB) at the University of Pennsylvania and were acknowledged by the Human Research Protection Office of the Department of Defense. All research, methods, and study protocols were conducted in accordance with the Human Subjects Institutional Review Board (IRB) at the University of Pennsylvania and the Human Research Protection Office of the Department of Defense. All participants provided informed consent before taking part in the study and were financially compensated. In Study 1, participants were compensated up to a total of $145. Compensation consisted of a $20 Amazon gift card to complete an online baseline survey; a $20 bonus Amazon gift card payment for at least 80% of the social group completing the survey; $50 for an in-person intervention training session, including an MRI scan session as part of a larger study (see Ref.^75^); and $55 for completing a 28-day EMA protocol with at least 70% response rate, compensated as Amazon gift card or cash card as per participant preference. In Study 2, participants were compensated $30 for completing an online survey and $60 for completing a 28-day EMA protocol via Amazon gift cards. Online surveys were conducted via Qualtrics, in-person tasks were presented using PsychoPy2, and the EMA prompts and participants’ responses were delivered via the LifeData app (www.lifedatacorp.com).

### Sample sizes

The target sample size for Study 1 (N = 240) was determined based on a power analysis accompanying an MRI session in the original grant application (see project protocol, Ref ^75^). However, recruitment was interrupted due to the COVID-19 pandemic, resulting in a final sample n = 108 when the study was closed for COVID reasons. For Study 2, our sample size was limited by the number of student groups originally recruited for Study 1 as part of a larger project (see Ref. ^75^), and all individuals who wished to enroll were included, yielding in a final sample of 218 individuals. See Supplementary Methods and Materials A for detailed information on participant enrollment and retention by condition.

### Participants

Study 1 sample comprised 108 individuals (65 female, 42 male, and 1 other/non-binary) recruited across two urban college campuses in the Northeastern United States. Participants were aged between 18 and 28 years (*M* = 20.54, S*D* = 1.70) and identified as white (53.7%), Asian (29.6%), Hispanic/Latino (4.6%), African American/Black (1.9%), and multiracial/other (10.2%). Study 2 sample comprised 218 individuals (157 female and 48 male; 13 did not respond) aged between 18 and 42 years (*M* = 20.6, *SD* = 2.1) recruited across two college campuses. Participants in Study 2 identified as white (37.2%), Asian (31.7%), African American/Black (7.8%), Hispanic/Latino (5.0%), multiracial/other (12.8%), and missing information (5.5%). In both studies, we recruited undergraduate students who were part of on-campus social groups (e.g., sports teams, arts groups, Greek life, etc.), with more than 80% of the group expressing interest in participation. See more information on eligibility criteria in the ‘Procedure’ section and in the Recruitment section of Supplementary Methods and Materials B. Diagrams of enrollment and retention can be found in Supplement A, Figs. S1 and S2.

### Procedure

More detailed study procedures can be found in the project protocol (see Ref. ^75^). Data collection for Study 2 replicated Study 1 but was modified to be completed fully remotely. In both studies, participants first completed an online, one hour long Qualtrics survey containing psychological, demographic and social network questionnaires on peer drinking behaviors, and other measures beyond the scope of the present report. Next, a subset of eligible participants were invited to attend an in-person (Study 1) session or to complete a virtual (Study 2) training session. In Study 1, eligibility for the intervention was determined by two components: a) the overall response completion rate of the social group and b) individual responses to questions in the baseline survey. Social groups were eligible to have their members invited to the intervention protocol if more than 20% of the group members completed the survey. Based on these criteria, 24 social groups were eligible. Of these groups, individuals were invited to complete the intervention protocol if they were: 18 years or older, had no history of serious medical issues, psychiatric hospitalization, or substance use disorders; reported drinking alcohol more than once a year; listed at least two people in their social group who drank the least in the group apart from themselves; were not studying abroad at the time, and reported being free from MRI contraindications including weighing less than 350 lbs; not claustrophobic, and not pregnant (See refs.^70,75–77^ for more information on an MRI session as part of the parent project). In turn, eligibility for Study 2 was open to any existing and new group members who completed any aspects of the broader project protocol. In Study 2, we excluded all individuals who had previously participated in the first intervention cohort. See Supplementary Methods and Materials B for more details.

Individuals who consented to take part in the intervention were randomized into three intervention conditions—mindfulness, perspective-taking, and control—, using the Qualtrics survey flow randomizer. As part of the intervention training session, participants underwent a brief instruction on how to respond to alcohol cues in daily life according to their respective condition. Details on the psychological distance training and the mindfulness instruction language development can be found in Supplement B. Training instructions for Study 1 and training videos for Study 2 are publicly available: https://osf.io/mpxws/.

The day following the in-person/online training, participants began a 28-day ecological momentary intervention and assessment protocol. Participants received twice-a-day reminders on how to respond to alcohol cues and responded to twice-a-day surveys measuring their alcohol use,  among other measures, for a total of 4 signals daily over 28 days. Intervention prompts with reminders on how to approach alcohol use, were sent at 2:00 pm and at 9:00 pm each day. Alcohol use surveys were sent at 8:00 and at 6:00 pm each day. See Fig. 1 for Study Procedure, and see Supplementary Methods and Materials B for more details on the EMA intervention protocol on the within-person reminder manipulation. Briefly, the two intervention conditions reinforced the active—mindfulness and perspective-taking prompts, respectively—for two weeks, and the control prompt for the other two weeks. We counterbalanced week order across participants (ABAB or BABA). Participants received no information about which weeks they were completing until they started the intervention protocol. We did not take additional steps to blind them to the study design.

### Measures

We used participant reports of drinking during the 28-day ecological momentary assessment period. Participants were asked: “Since your EVENING/MORNING survey, have you consumed any alcohol? (“No” or “Yes” response option). Participants who responded “Yes”, were asked to enter the number of standard servings of beer, liquor, and wine consumed since the previous survey using a numeric entry. Responses for each beverage category were summed to obtain the total servings of alcohol consumed for each assessment (see Ref.^51^ for previous use of these scales to capture alcohol use during daily life). When participants responded “No” to having consumed alcohol, they answered questions about physical activity, caffeine use, and water consumption. These questions were matched for length with the follow-up alcohol questions to reduce the possibility that participants would report no alcohol use in order to minimize survey completion time. To select low drinking peer targets for participants randomized in the perspective-taking condition, we used peer nominations from the baseline social network questionnaire in response to: “Who in your group drinks the least?”. In cases when individuals nominated more than one peer who drinks less than them, targets were selected by picking a peer who was also nominated as close (“Who are you closest to?”) and who reported the highest drinking difference from the individual.

### Data preparation

Drinking was defined as the number of total alcohol servings consumed at each assessment over the 28-day period. In Study 1, the three largest, improbable values of drinks per occasion (24, 36, 60) were winsorized to the next largest value—16 drinks per occasion. This step applied to 7 signals across 3 individuals out of 5492 observations in total. No outliers were observed in Study 2.

### Data analysis

To account for the nature of the alcohol use data which are often positively skewed and include many observations at zero, we used multilevel hurdle models. Hurdle models include a logistic regression to model the zeroes in the data as well as a count regression (in this case negative binomial) to model the counts. All the zeroes (not alcohol use occasions) are modeled with the logistic regression and nonzero-counts (alcohol use occasions) are modeled by a truncated negative binomial (i.e., truncated as it does not contain zero). Thus, these models allowed us to independently model whether a person drinks or not (logistic regression) at a given occasion and number of drinks when an individual drinks (count regression). We estimated hurdle models^78^ using glmmTMB^79^ in R (Version 4.0.3) using the RStudio interface (Version 1.3.1093).

We specified two models (one for Study 1 and another for Study 2) to assess whether drinking frequency and amount vary among participants in the mindfulness and perspective-taking conditions (within-person) on weeks when intervention reminders were present or absent. The main predictor of interest was active vs. inactive week and the outcomes were frequency of drinking occasions and drinking amount. To further explore if the effectiveness of the reminders varied by condition type (mindfulness vs. perspective-taking), we included an interaction term: active week (vs. inactive week) x condition type. Given that observations are nested within participants, who are in turn nested within non-independent social groups, all multilevel models accommodated the nested nature of the data, and intercepts were allowed to vary randomly across people. In models presented in the main manuscript, we controlled for the following covariates: social weekend (defined as Thursday, Friday, or Saturday), due to differences between weekend and weekday drinking among college students^80^; participant response rates, given observed differences in response rates on active versus inactive weeks; and signal count given overall decreases in drinking over time throughout the study period. Additional models controlling for demographic covariates are presented in the Supplement C (Table S2). We observed similar results when controlling for age, gender and race. All results remained robused when removing covariates.

### Follow-up behavior change analyses

To explore whether the main findings, i.e., changes in drinking occasion frequency from active to inactive weeks differed from non intervention-related changes, we compared drinking frequency change scores among participants in the interventions and the control condition. For each person, we calculated a change score that captures differences in the average proportion of drinking occasions on active intervention weeks versus inactive weeks throughout the 28-day study period. First, we first calculated the average proportion of drinking occasions on active weeks and inactive weeks, separately, by dividing the number of drinks reported throughout the active and inactive intervention period by an individual’s number of responses to alcohol reminders during the same time period. Next, we subtracted the proportion of drinking occasions on active weeks from the proportion of drinking occasions on inactive weeks.

To obtain change scores for participants in the control condition, who did not undergo a within-person active-to-inactive week manipulation as part of the protocol, we randomly assigned two of the four weeks in the study period as pseudo “active” and the remaining two as pseudo “inactive” weeks. To counterbalance week order, as done in the active treatment groups, we randomly split half of the participants in an ABAB design, or a BABA design, which allowed us to match the two intervention designs as closely as possible. We repeated this random assignment 100 times to stabilize the estimates. Next, we averaged the change scores in drinking frequency (from pseudo-inactive to pseudo-active weeks) for the control participants across all iterations and compared the control group’s pseudo change scores to the intervention groups’ change scores. We first checked whether the proportion of drinking occasions were significantly non-Gaussian using the Shapiro Wilk test of normality and chose to perform non-parametric Mann–Whitney–Wilcoxon tests instead of t-tests because of nonnormality. We performed this comparison using unpaired two-sided Wilcoxon tests.

### Author identities

Mindful that our identities can influence our approach to science, the authors wish to provide the reader with information about our backgrounds. With respect to gender, when the manuscript was drafted, nine authors self-identified as women, five as men, and one as non-binary. With respect to race, 13 authors self-identified as White, one as Asian, and one as Black. With respect to engagement with college students, when this study was conducted, two were doctoral students who teach and/or mentor other students, one was a research coodinator who mentors other students, five were postdoctoral researchers or research scientists who teach and/or mentor students, and seven were professors who teach and/or mentor students.

## Supplementary Information

Supplementary Information.

## Data Availability

De-identified data are available on Github: https://github.com/miajov/psych-distance-intervention.

## Code availability

Code to reproduce the main analyses is available on Github: https://github.com/miajov/psych-distance-intervention.
