# Supplementary material for: Psychological distance intervention reminders reduce alcohol consumption frequency in daily life
Source: Sci Rep. 2023 Jul 25;13:12045. doi: 10.1038/s41598-023-38478-y (PMC10368637; doi:10.1038/s41598-023-38478-y)
Supplement: Supplementary file 1 — Supplementary Information. [file 41598_2023_38478_MOESM1_ESM.docx]

**Supplementary information**

The following documents contains supplementary information for Jovanova *et al*., Psychological distancing reminders reduce drinking frequency in daily life.

Supplement A presents information on participant recruitment, retention, and study procedures. Supplement B includes information on intervention training and pilot data collected to help develop the mindfulness language. Supplement C includes descriptive statistics, sensitivity analyses that account for demographic covariates and outliers, and robustness checks. Supplement D includes additional between-person analyses.

**Supplementary Methods and Materials A**


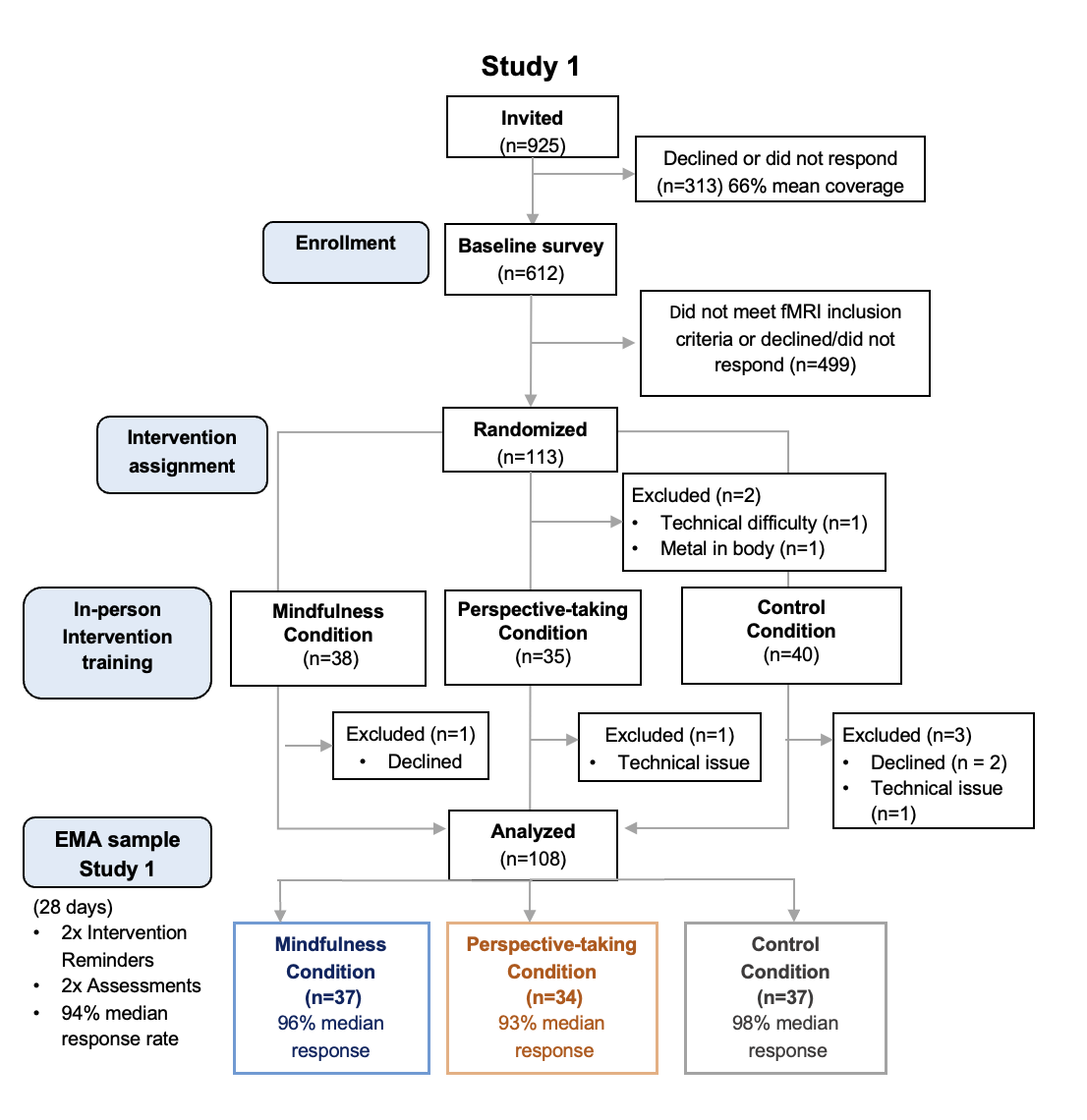


**Fig S1. Participant enrollment and retention in Study 1.**


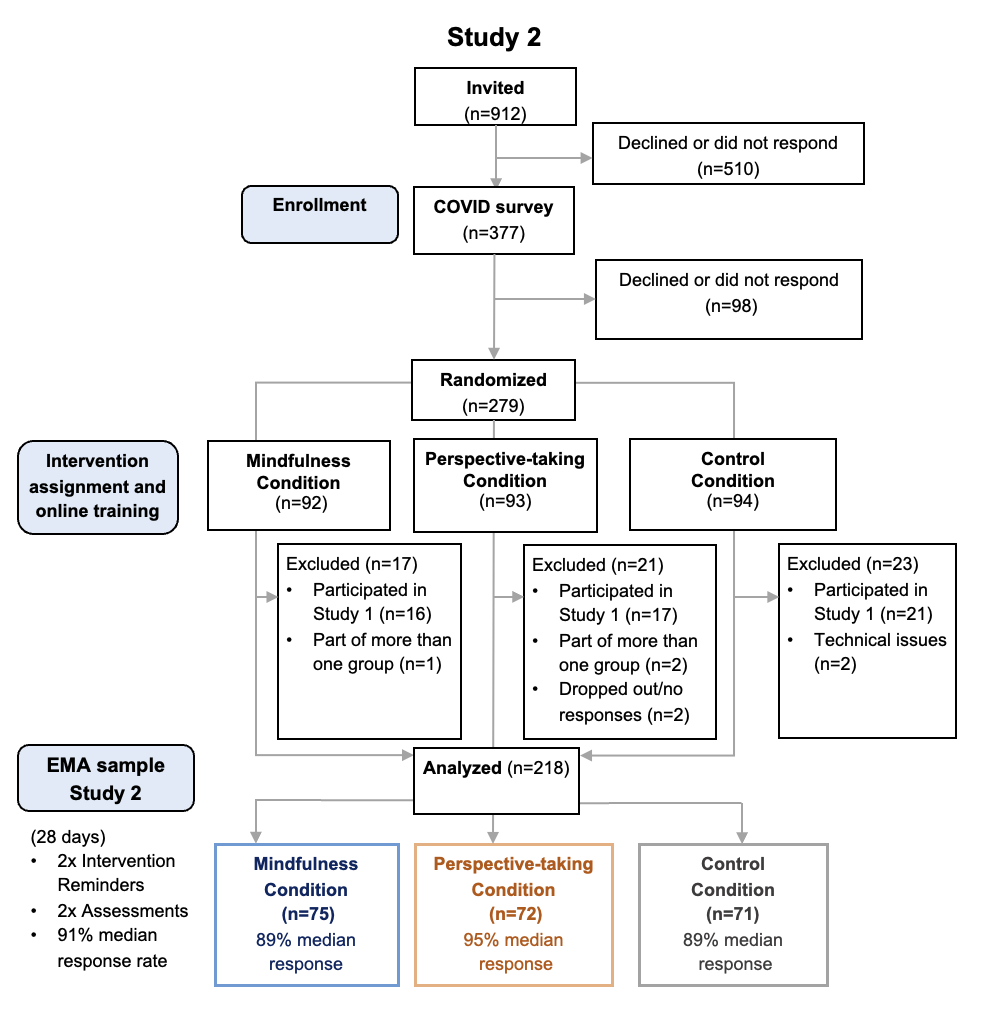


**Fig S2. Participant enrollment and retention in Study 2.**

**Supplementary Methods and Materials B**

The following section on methods and study procedures is in part reproduced and adapted from the parent project protocol (Ref[^1^](https://paperpile.com/c/X7ucKN/g6J0f)). The language is adapted for ease of reader comprehension.

**Recruitment**

Recruitment materials advertised a study titled “Social Health Impact of Network Effects Study (SHINE)” to undergraduate students who were members of on-campus social groups across two urban, Northeastern universities in the United States. In Study 1, eligible social groups included on-campus organizations (i.e. social clubs or sports teams) of students with 20-100 members, with at least 80% of the members interested in participating in the study. The study was advertised through print flyers, university websites, email communication, and information sessions. To reach students, researchers first contacted social group leaders to inform them of the study and to assess group interest in attending an in-person, digital, or paper information session. Groups who expressed interest were offered a 5-minute information session where researchers answered questions about the study and shared informational handouts with group members. Additionally, researchers contacted student housing directors and professors, across Marketing, Psychology and Communication departments, via a study email account to share online and print study flyers in student dorms and classes. Next, we employed a snowball sampling approach, such that interested students could share study information with their peers who were members of additional on-campus social groups. Student groups who were interested in participating were asked to email the study account in response to flyers, informational sessions, email advertisements, and word of mouth. Next, all interested students were asked to complete an initial online eligibility survey.

In Study 1, of the 1024 individuals in the eligible social groups identified by the study team, 925 individuals stated that they were interested in potentially participating via email and were invited to take the online baseline survey. These individuals were from 24 social groups across the two universities (33% performing arts groups, 29% sororities or fraternities, 25% sports clubs, 8% technology clubs, 4% other). Participants who expressed further interest after the initial invite (*N* = 612; 59% of invited participants) consented to participate and completed a baseline survey. At baseline, participants (*N* = 587 from 24 groups) completed an hour-long online survey that characterized their social networks and assessed MRI eligibility, baseline alcohol use, demographics, as well as individual responses to a number of different questionnaire measures, beyond the scope of the current study. An additional 25 participants who enrolled in the study at a later point completed an abbreviated baseline survey in conjunction with the COVID assessment, yielding a total of *N* = 612.

In Study 1, eligibility for the alcohol intervention was determined by two components: a) the overall response completion rate of the social group and b) individual responses to questions in the baseline survey. Social groups were eligible to have their members invited to the intervention protocol if more than 20% of the group members completed the survey. Based on these criteria, 24 social groups were eligible. Of these groups, individuals were invited to complete the intervention protocol if they were: 18 years or older, had no history of serious medical issues, psychiatric hospitalization, or substance use disorders; reported drinking alcohol more than once a year; listed at least two people in their social group who drank the least in the group apart from themselves; were not studying abroad at the time, and reported being free from MRI contraindications including weighing less than 350 lbs; not claustrophobic, and not pregnant (See refs.[^1^](https://paperpile.com/c/X7ucKN/g6J0f)^,^[^2–4^](https://paperpile.com/c/X7ucKN/kc5K2+jpyGr+o0AP) for more information on an MRI session as part of the parent project). Of the 113 eligible participants who completed the baseline survey and the MRI session; five individuals were excluded from the alcohol intervention protocol for declining to participate (n =3) or technical issues (n =2), for a total of 108 final participants included in analyses. See Figure S1 for exclusion by condition. Although we initially planned to enroll a larger sample in the intervention component, in-person data collection was terminated in March 2020 due to the COVID-19 pandemic. The data collection for Study 1 began on February 2^nd^, 2019 and ended on March 11^th^, 2020 for 87% of the sample (n = 94). The remaining 13% (14 participants) completed data collection by April 7^th^ , 2020 thus including time primarily on campus and prior to the pandemic, and several responses while transitioning home at the start of the COVID-19 pandemic.

Eligibility for Study 2 was open to any existing and new group members who completed any aspects of the broader project protocol, and we applied no exclusion criteria. Participants who had completed any component of the study, as well as new members from the social groups were invited to participate and 377 individuals consented to complete an initial COVID baseline survey. The COVID-baseline characterized their social networks, assessed demographics, as well as individual responses to a number of different questionnaire measures, beyond the scope of the current study. We expanded the opportunity to complete the 28-day EMA intervention protocol to all participants who completed the COVID survey. A total of 279 participants enrolled in the EMA component. Of these participants, we excluded 61 participants from analyses for: participating in the intervention cohort in Study 1(n = 54) , duplicate entries (n=3); drop out (n=2), and technical issues (n=2), resulting in a total of 218 participants included in final analyses. See Figure S2 for exclusion by condition. Data collection for Study 2 began on May 30, 2020 and ended on October 27, 2020.

**Psychological distance training**

In the next section, we provide details about the intervention training. We explain Study 1 first and Study 2 second. Following the online baseline survey, participants were invited to complete a brief introductory training on how to approach alcohol cues encountered in everyday life, either in-person (Study 1) or online (Study 2). Consistent with past work on regulation of alcohol craving, participants viewed images of alcohol (beer, wine, and liquor) to elicit craving[^5^](https://paperpile.com/c/X7ucKN/w9vdm) and were trained to respond to these alcohol cues using different regulation strategies according to their respective condition: mindfulness, perspective-taking, and control. In Study 1, a researcher guided participants through the instructions on how to respond to alcohol on a computer screen in the lab. The training consisted of two blocks of four trials, for a total of ~12 seconds per trial. Each block began with a condition instruction cue (3s), followed by an alcohol image cue (6s) and a craving rating (3s), to parallel a MRI task on alcohol cue regulation beyond the scope of the current report[^1^](https://paperpile.com/c/X7ucKN/g6J0f). Researchers checked for comprehension of the instructions verbally. In Study 2, participants viewed standardized training videos on how to respond to alcohol on their mobile devices (~1.65min long on average), closely matching the instructions in Study 1. See Intervention training components for more details on instructions by condition. To verify comprehension, participants were required to pass comprehension checks on their personal devices via Qualtrics during the training. Researchers examined the responses and verified participant comprehension of the training. To allow for reproducibility, we make the instruction materials from Study 1 and the training videos from Study 2 publicly available: https://osf.io/mpxws/.

**Intervention** **training components**

**Mindfulness.** Participants viewed images of alcohol beverages paired with text instructions, piloted across 14 Mechanical Turk studies prior to the study (See Mindfulness piloting below). The text instructed participants to either approach the alcohol image mindfully, by mentally taking a step back in order to observe the situation and their responses in an impartial and non-judgmental manner or to react naturally. During mindfulness trials, participants were further asked to pay attention to their reaction without getting caught up in it. In contrast, on control trials, participants were asked to approach the alcohol image as they normally would approach alcohol in their daily life, by reacting naturally or to have whatever thoughts and feelings they would normally have. By alternating mindful and natural responses to alcohol stimuli, the training intended to instruct participants to differentiate between adopting a mindful vs. typical response to alcohol in daily life.

**Perspective-taking.** Participants in the perspective-taking condition were presented with an image of an alcoholic beverage paired with instructions to either respond to the image from the perspective of their two lowest-drinking peers or naturally from their own perspective. Specifically, on perspective-taking trials, a peer’s name was prompted on the screen, and participants were asked to imagine the thoughts and feelings their peer would have when approaching the alcohol cue in the same situation. Peer names were selected based on the participant's nominations of group members whom they reported to drink the least, and with whom they were closest to, based on responses in the baseline survey. On control trials, participants were asked to approach alcohol from their own perspective. By alternating peer- and self-focused responses to alcohol stimuli, the training intended to help participants differentiate between taking their peers’ and their own perspective when encountering alcohol cues.

**Control.** The third group completed the same task without any regulatory strategy suggested. All participants viewed images of alcoholic beverages (e.g., bottle of beer), and were asked to respond naturally as they typically would in their everyday life.

**Ecological momentary intervention study entry**

Following the in-lab (Study 1) and online (Study 2) psychological distancing training, participants were invited to participate into a 28-day ecological momentary intervention and assessment period. In Study 1, researchers walked through participants through the protocol and installation of the LifeData app ([www.lifedatacorp.com](http://www.lifedatacorp.com))— used to collect alcohol use responses on personal mobile devices—at the end of the in-person session. In Study 2, participants received an email invitation link at the end of the baseline survey with a possibility to opt into the intervention protocol. Interested participants received email instructions to download the LifeData app and completed an online comprehension quiz assessing their understanding of the intervention protocol instructions. The intervention instructions walked participants through the definitions of a “standard serving” of different types of drinks; explained the general prompts and kind of questions they’d receive from the LifeData app and at what times in the next month; and asked them to try to answer the survey questions promptly. Example comprehension questions asked: “In your own words, what are you supposed to do when you receive an instruction to be mindful/react naturally/take your peer’s perspective in this study?”. Further, participants in the perspective-taking condition were noted of their study peer’s name for the purpose of the study. In both studies, participants who consented to participate in the intervention component were randomly assigned to conditions using the Qualtrics survey flow randomizer. Participation in the EMA protocol began the day following the in-person session (Study 1) and following the online study instruction set up (Study 2).

**Ecological momentary intervention protocol**

Participants received two intervention prompts with reminders on how to approach alcohol use, one at 2:00 pm and the second at 9:00 pm each day over 28 days. The content of the prompts varied according to the three assigned conditions: the control message stated “If you are around alcohol today, REACT NATURALLY — have whatever thoughts and feelings you would normally have”; the mindfulness message stated “If you are around alcohol today, REACT MINDFULLY — notice, acknowledge, and accept the thoughts and feelings you have”; the perspective-taking message stated “If you are around alcohol today, IMAGINE HOW [NAME] WOULD REACT — try to imagine the thoughts and feelings that [NAME] would have”, where [NAME] contained the name of their lowest-drinking study peer.

*Within-person manipulation.* Participants in the control condition received the control/REACT NATURALLY prompt consistently over the four weeks and did not partake into a within-person manipulation. In turn, the two intervention conditions reinforced the active — mindfulness and perspective-taking prompts, respectively— for two weeks, and the control prompt for the other two weeks. We counterbalanced week order across participants (ABAB or BABA), allowing for a within-person manipulation. Participants received no information about which weeks they were completing until they started the intervention protocol. We did not take additional steps to blind them to the study design.

**Ecological momentary assessment**

In addition to the twice-a-day intervention prompts, participants answered two daily surveys. A morning survey was sent at 8:00 am and an evening survey was sent at 6:00 pm. The surveys assessed alcohol consumption, and additional measures not the focus of the present report such as mood, conversations about alcohol, emotion regulation, and others.

**Mindfulness piloting**

To inform the language used in the main intervention training, we explored nine different variations of mindfulness-related instructions. We conducted a series of online studies via Amazon’s Mechanical Turk (total n =700). Consistent with past work on alcohol regulation, participants viewed images of alcohol (beer, wine, and liquor[^5^](https://paperpile.com/c/X7ucKN/w9vdm)) paired with manipulated instructions across different trials and rated their alcohol craving on a scale from 1-5 after each trial. In each pilot, participants viewed one of 9 different versions of mindfulness instructions on some trials and control instructions on other trials. Three of the pilots emphasized instructions related to psychological distancing, whereas the remaining versions emphasized different components, such as attention to the present moment, focus, awareness, or acceptance and contained no distancing language.

We ran multilevel models using lmer[^6^](https://paperpile.com/c/X7ucKN/CqqIe) in R (Version 4.0.3) using the RStudio interface (Version 1.3.1093) to compare craving ratings on trials after participants viewed manipulated intervention instructions vs. control instructions. These data suggest a trend such that instruction trials that emphasized psychological distancing (Fig S3A, replicated in B and C) directionally decreased alcohol craving vs. instruction trials which instead emphasized other components of mindfulness (Fig S3D–I). While more experimental work is needed, these data suggest that psychological distancing may be an important mechanism to change responses to alcohol cues and down-regulate craving. See Fig S4 for one of the nine example instructions without distancing and other instructions that include distancing (as well as the final instructions used in the project).


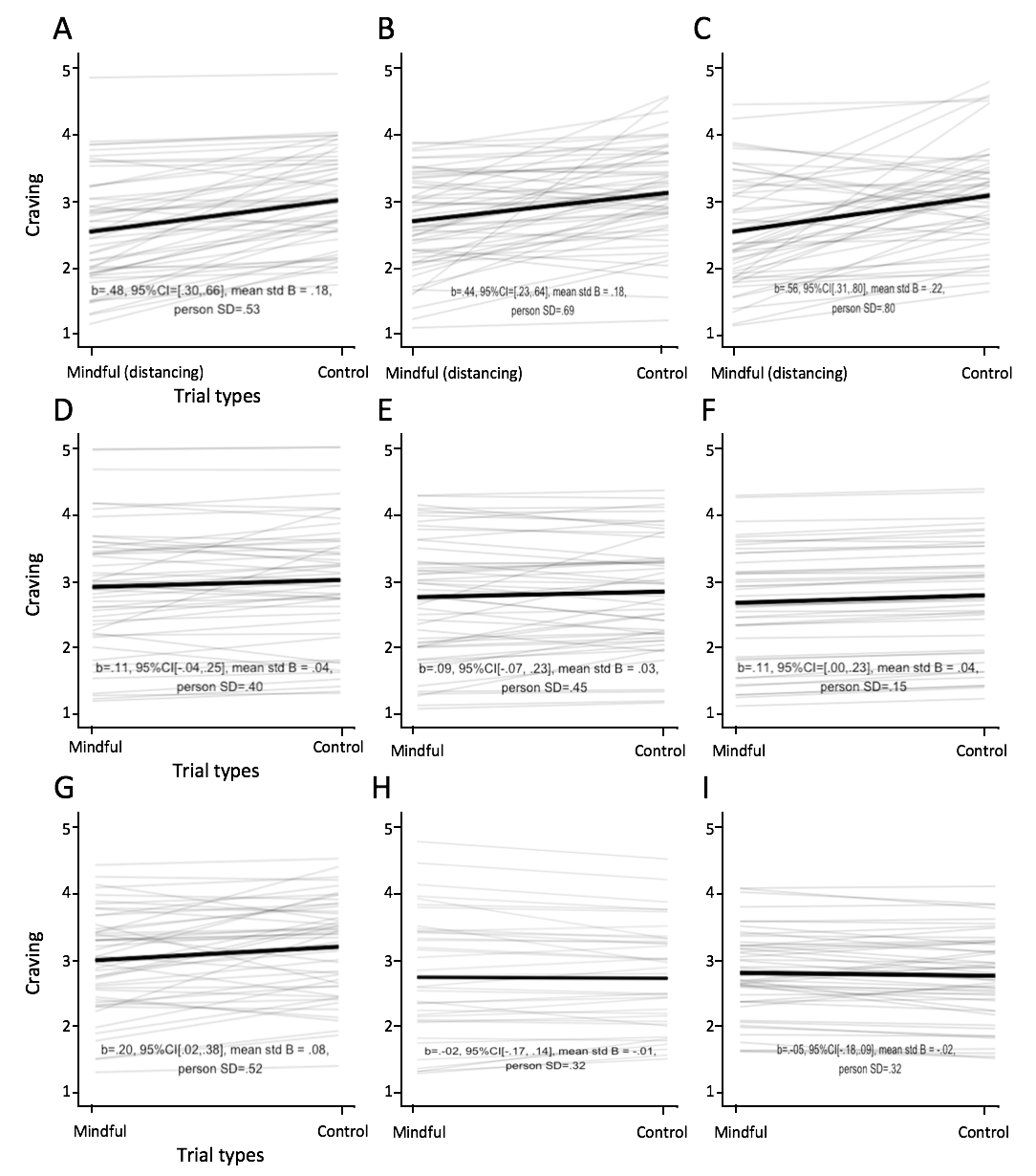


**Fig S3. Effects of different mindfulness instructions on alcohol craving across instruction versions with varying emphasis on psychological distancing from alcohol cues**. Across this series of pilot studies, the data indicate that mindfulness instructions emphasizing distancing (A-C) may be more effective in reducing craving than instructions emphasizing other components of mindfulness (D-I). Further, mindfulness instructions that contained no distancing language had near zero effect on reducing craving.

| **Mindfulness instruction with distancing (Final version used in the current project)**  Another way you can relate to these situations is by *mentally taking a step back* in order to observe the situation and your response to it in an impartial and nonjudgmental manner.  You may simply notice your thoughts and feelings about these situations, perhaps with some curiosity. That way, you can actively pay attention to your reaction and *see it as just a passing pattern of thoughts and feelings, without getting caught up in it.*  If you see a picture of beer, you can *mentally distance yourself* by observing the situation, and your response to it, with a more impartial, nonjudgmental, or curious mindset. When you see the word MINDFUL, it is critical that you *mentally take a step back from the situation, so that you observe the situation and your response to it without getting caught up in it.* |
| --- |
| **An example mindfulness instruction without distancing**  Another way you can relate to these situations is by *actively noticing how they make you feel* in order to observe the situation and your response to it in an impartial and nonjudgmental manner. You may simply notice your thoughts and feelings about these situations, perhaps with some curiosity. That way, you can actively pay attention to your reaction and *observe your thoughts and feelings without judging your response.*  If you see a picture of beer, you can *notice any thoughts, sensations, and cravings or lack of cravings that arise.* When you see the word MINDFUL, it is critical that you *look at the photo and simply notice and observe any sensations you experience, so that you simply attend to what is felt, without making any judgment of the "goodness" or the "badness" of that sensation.* |

**Fig S4.**  Examples of pilot mindfulness instructions with or without distancing-related language.

**Supplement C. Table S1** *Descriptive statistics across interventions.*


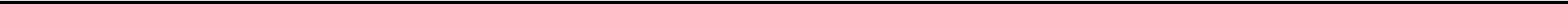


**Study 1**


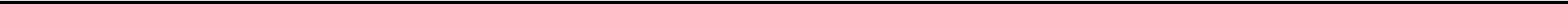


| **Condition** |  |  | Mindfulness | |  |  |  | Perspective-taking | | |  |  |  | Control |  |  |  |
| --- | --- | --- | --- | --- | --- | --- | --- | --- | --- | --- | --- | --- | --- | --- | --- | --- | --- |
|  |  |  |  |  |  |  |  |  |  |  |  |  |  |  |  |  |  |
| Intervention | Active weeks | | | Inactive weeks | | | Active weeks | | | Inactive weeks | | |  | N/A |  |  |  |
| Week |  |  |  |  |  |  |  |  |  |  |  |  |  |  |  |  |  |
|  |  |  |  |  |  |  |  |  |  |  |  |  |  |  |  |  |  |
|  |  |  |  |  |  | |  |  |  |  |  |  |  |  |  |  |  |
|  | *M* | *M_win* | *Median* | *M* | *M_win Median* | | *M* | *M_win* | *Median* | *M* | *M_win* | *Median* | *M* | *M_win* | *Median* |  |  |
|  |  |  |  |  |  |  |  |  |  |  |  |  |  |  |  |  |  |
| % EMA | 89.96% | 89.96% | 96.43% | 91.22% | 91.44% 96.43% | | 86.45% | 86.87% | % | 92.54% | 93.20% 96.43% | | 92.86% | 94.81% 98.21% | |  |  |
| responses |  |  |  |  |  |  |  |  |  |  |  |  |  |  |  |  |  |
|  |  |  |  |  |  |  |  |  |  |  |  |  |  |  |  |  |  |
| % Drinking | 12.63% | 10.3% | 10.71% | 15.6% | 14.63% | 14.29% | 12.95% | 8.8% | 9.56% | 15.53% | 14.3% | 7.28% | 17.61% | 14.82% | 14.55% |  |  |
| occasions |  |  |  |  |  |  |  |  |  |  |  |  |  |  |  |  |  |
|  |  |  |  |  |  |  |  |  |  |  |  |  |  |  |  |  |  |
| Drinks per | 2.61 | 2.34 | 2.29 | 2.87 | 2.77 | 2.69 | 2.92 | 2.60 | 2.86 | 2.98 | 2.95 | 2.90 | 3.56 | 2.78 | 3.1 |  |  |
| occasion |  |  |  |  |  |  |  |  |  |  |  |  |  |  |  |  |  |
|  |  |  |  |  |  |  |  |  |  |  |  |  |  |  |  |  |  |
|  |  |  |  |  |  |  |  |  |  |  |  |  |  |  |  |  |  |
|  |  |  |  |  |  |  | **Study 2** |  |  |  |  |  |  |  |  |  |  |
|  |  |  |  | |  |  |  |  | | |  |  |  |  |  |  |  |
| **Condition** |  |  | Mindfulness | |  |  |  | Perspective-taking | | |  |  |  | Control |  |  |  |
|  |  |  |  |  |  |  |  |  |  |  |  |  |  |  |  |  |  |
| Intervention | Active weeks | | | Inactive weeks | | | Active weeks | | | Inactive weeks | | |  | N/A |  |  |  |
| Week |  |  |  |  |  |  |  |  |  |  |  |  |  |  |  |  |  |
|  |  |  |  |  |  |  |  |  |  |  |  |  |  |  |  |  |  |
|  |  |  |  |  |  | |  |  |  |  |  |  |  |  | |  |  |
|  | *M* | *M_win* | *Median* | *M* | *M_win Median* | | *M* | *M_win* | *Median* | *M* | *M_win* | *Median* | *M* | *M_win Median* | |  |  |
|  |  |  |  |  |  |  |  |  |  |  |  |  |  |  |  |  |  |
| % EMA | 81.48% | 81.09% | 89.29% | 85.33% | 91.94% 96.43% | | 78.57% | 77.92% 92.86% 82.79% | | | 92.55% 94.64% | | 80.53% | 88.78% 89.29% | |  |  |
| responses |  |  |  |  |  |  |  |  |  |  |  |  |  |  |  |  |  |
|  |  |  |  |  |  |  |  |  |  |  |  |  |  |  |  |  |  |
| % Drinking | 9.14% | 5.82% | 3.71% | 12.08% | 8.91% | 7.41% | 9.33% | 6.14% | 3.70% | 10.77% | 7.89% | 3.70% | 9.87% | 7.69% | 4.76% |  |  |
| occasions |  |  |  |  |  |  |  |  |  |  |  |  |  |  |  |  |  |
|  |  |  |  |  |  |  |  |  |  |  |  |  |  |  |  |  |  |
| Drinks per | 2.31 | 1.87 | 1.70 | 2.34 | 2.02 | 2.25 | 1.93 | 1.86 | 1.92 | 1.72 | 1.57 | 1.50 | 1.94 | 1.07 | 1.82 |  |  |
|  |  |  |  |  |  |  |  |  |  |  |  |  |  |  |  |  |  |

occasion


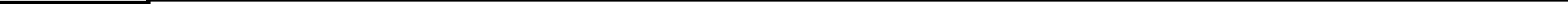


**Note.** *M* = raw means; *M_win* = winsorized means (winsorized to +/- 2 standard deviations).

**Supplementary Analyses C**

In the subsequent section, we provide results from robustness checks and sensitivity analyses. First, we conducted two multilevel hurdle models, one for Study 1 and another for Study 2 to examine if the within-person effects of the psychological distance reminders on drinking frequency, presented in the main manuscript, remain robust when controlling for additional demographic variables. To test this, we ran parallel multilevel models to those presented in the main manuscript, but we also included gender, age, and race as covariates. Next, we explored whether the between-person effects on changes in drinking frequency, presented in the main manuscript, are robust to possible effects of outliers. To explore this, we ran parallel tests to those presented in the main manuscript, winsorizing outliers to +/- 2 standard deviations from the mean.

**Psychological distance reminders reduce drinking frequency controlling for demographics.** We observed consistent effects in both studies when controlling for age, gender, and race. Within-person, we observed lower drinking frequency on active weeks when individuals were instructed to adopt a mindful approach or to take their lower drinking peer’s perspective, relative to inactive weeks, when they received control prompts, which instructed them to respond to alcohol naturally (Study 1: OR = 1.34, 95% CI [1.01-1.77], *p* = .042); Study 2: OR = 1.34, 95% CI [1.04 – 1.74], *p* = .025; Table S2). Consistent with results presented in the main manuscript, we observed no significant intervention effects on the number of drinks when drinking, and no week (active vs. inactive) x condition (mindfulness vs. perspective-taking) interaction effects in either study when accounting for demographics (Table S2).

**Psychological distance reminders reduce drinking frequency vs. control.** As a robustness check of the potential influence of outliers, we repeated the same analyses presented in the main manuscript comparing changes in drinking frequency between the intervention groups (change from inactive to active weeks) and the control group (change from pseudo-active to pseudo-inactive weeks). However, here we winsorized drinking frequency change scores +/- 2 standard deviations from the mean. Consistent with the finding reported in the main text, we found significant differences in behavior change between the two groups such that individuals in the intervention groups showed a greater decrease in drinking frequency from inactive weeks to active weeks relative to the control group, both in Study 1 (*W* = 984.5, *Z* = -2.146, *p* = .032, *r* = .206) and in Study 2 (*W* = 3750.5 *Z* = -3.475,  *p* =.001, *r* = .235; Fig. 3). Together, these results reduce the likelihood that the observed between-person differences in behavior change, i.e., decrease in drinking frequency, between the intervention and control groups is driven by outliers.

**Table S2. Within-person effects of reminders on drinking on active vs. inactive weeks controlling for demographic variables**

|  | | **Study 1** | | | | | | | | | **Study 2** | | | | | | | |  |
| --- | --- | --- | --- | --- | --- | --- | --- | --- | --- | --- | --- | --- | --- | --- | --- | --- | --- | --- | --- |
|  | | **Zero-inflated sub-model** | | | | | | | | | | | | | | | | |  |
| Fixed Effects | | OR | | 95%CI | | | | | *p* | | OR | | 95%CI | | | | *p* | |  |
|  | Intercept  Active week (vs. inactive)  Perspective condition (vs. mindful)  Signal count  Number of responses  Social weekend (vs. week)  Age  Gender  Race  Perspective condition (vs. mindful) *active week (vs. inactive) | | 59.6  1.34  1.12  1.01  1.03  0.50  0.85  0.91  0.98  0.93 | | [1.34-2653]  [1.01-1.77]  [0.6- 1.89]  [1.01-1.02]  [0.99-1.06]  [0.40-0.61]  [0.74-0.98]  [0.55-1.48]  [0.81-1.19]  [0.62-1.41] | | | | | .004  .042  0.67  <.001  .165  001  .028  690  .838  .743 | | 103  0.34  0.96  1.00  1.01  0.53  0.89  1.50  0.91  1.05 | | [0.74-14,349]  [1.04-1.74]  [0.55-1.67]  [0.99-1.01]]  [0.99-1.04]  [0.44-0.64]  [0.71-1.10]  [0.69-3.25]  [0.75-1.11]  [0.72-1.54] | | | | .066  .025  .890  .991  .324  <.001  .280  .305  .357  .783 | |
| Random effects | | Variance | | | | SD | | | | | Variance | | | | SD | | | |  |
| Intercept  Participant ID* Group ID  Participant ID | | <.011  <.001 | | | | .902  <.001 | | | | | 1.599  .781 | | | | 1.265  .884 | | | |  |
|  | | **Conditional sub-model** | | | | | | | | | | | | | | | | |  |
| Fixed Effects | | OR | | 95%CI | | | | | *p* | | OR | | 95%CI | | | | *p* | |  |
| Intercept  Active week (vs. inactive)  Perspective condition (vs. mindful)  Signal count  Number of responses  Social weekend  Age  Gender  Race  Perspective condition (vs. mindful) *active week (vs. inactive) | | 9.89  0.94  1.04  1.00  0.98  1.56  0.99  0.76  0.95  1.07 | | [0.58-167]  [0.70-1.26]  [0.71-1.54]  [0.99-1.00]  [0.96-1.01]  [0.26-1.93]  [0.89-1.09]  [0.54-1.09]  [0.83-1.08]  [0.71-1.61] | | | | | 0.112  .669  .828  .184  .169  <.001  .778  .141  .414  .758 | | 161  0.95  0.48  1.01  0.99  1.32  0.84  .62  .95  1.41 | | [9.27-2,793]  [0.75, 1.22]  [0.34, 0.68]  [1.00, 1.01]  [0.98, 1.01]  [1.09, 1.61]  [0.74, 0.96]  [0.43, 0.90]  [0.86, 1.05]  [0.96, 2.08] | | | | <.001  .699  <.001  .061  .302  .005  .009  .013  .314  .076 | |  |
| Random effects | | Variance | | | | | SD | | | | Variance | | | | SD | | | |  |
|  | Intercept  Participant ID* Group ID  Participant ID | | .205  .033 | | | | | .453  .182 | | | | .1569  .058 | | | | .396  .241 | | | |

**Note.** Study 1: 3577 observations nested within 71 participants across 10 groups; Study 2: 6301 observations nested within 138 participants across 23 groups. The zero-inflation sub-model of the hurdle model estimates the probability of an extra zero (no alcohol use) such that a positive estimate indicates a higher chance of no alcohol use. Perspective = perspective-taking; Mindful = mindfulness.

**Supplementary Analyses D**

We conducted follow-up analyses to examine group differences between the intervention groups, on active weeks, relative to the control group. This comparison tests whether drinking levels on active intervention weeks differs from drinking levels among individuals who only received non-intervention (control) reminders throughout the entire protocol. We conducted two multilevel hurdle models, one for Study 1 and another for Study 2, examining effects on drinking occasion frequency and drinking amount. We controlled for the same covariates as those reported in the main manuscript (see Data Analysis section in the main manuscript): gender, age, and race, as well as baseline drinking amount and frequency where available in Study 1.

We found no differences in the frequency of drinking occasions on active weeks (among intervention groups) versus the control group across both studies: Study 1 (mindfulness vs. control: OR =1.28, 95% CI [0.86-1.91], *p* =.229; perspective-taking vs. control: OR =1.02, 95% CI [0.67-1.67], *p* =.923; Study 2 (mindfulness vs. control: OR = 1.18, 95% CI [0.65- 2.13], *p* = .583; perspective-taking vs. control: OR = .97, 95% CI [0.52 - 1.80], *p* = .928; Table S3). In terms of group differences in drinks per drinking occasion, individuals in the perspective-taking group had fewer drinks per occasion (OR = .69, 95% CI [0.50 - 0.96], *p* = .028; Table S4) in Study 1, however, we did not replicate this effect in Study 2 (perspective-taking vs. control: OR = .97, 95% CI [0.52 - 1.80], *p* = .928; Table S4). We observed similar number of drinks per occasion between the mindfulness vs. control group (Study 1: OR = 1.36, 95% CI [0.91 - 2.02], p = .134; Study 2: OR = .96, 95% CI [0.63 - 1.46], *p* = .839; Table S4). These results indicate that even though the intervention groups drank less frequently on active vs. inactive weeks at the within-person level (Table 1), the intervention groups did not drink less often on active weeks compared to the control group at the between-person level (Table S3). It is possible that there may have been pre-existing group differences in alcohol use that were not overcome by random assignment, and therefore it is important to also evaluate effects within-person, which is ultimately the intervention target.

**Table S3. Group differences in drinking occasion frequency: active weeks .vs control group**

|  |  |  | **Study 1** | |  |  | **Study 2** | |  |  |
| --- | --- | --- | --- | --- | --- | --- | --- | --- | --- | --- |
|  |  |  |  | | | |  |  |  |  |
|  | Fixed Effects | | OR | 95%CI | | *p* | OR | 95%CI | | *p* |
|  |  |  |  |  | |  |  |  | |  |
|  |  | Intercept | 1038.94 | [95.57 – 11294.6] | | <.001 | 119.37 | [5.11 – 2789.88] | | .003 |
|  |  | Mindful condition (vs. control) | 1.27 | [.87 – 1.86] | | .211 | 1.18 | [0.65 – 2.13] | | .583 |
|  |  | Perspective condition (vs. control) | 1.15 | [.76 – 1.75] | | .510 | 0.97 | [0.53 – 1.80] | | .928 |
|  |  | Signal count | 1.02 | [1.01 – 1.02] | | <.001 | 1.00 | [0.99 – 1.01] | | .836 |
|  |  | Number of responses | 1.02 | [.99 – 1.04] | | .139 | 1.01 | [0.99 – 1.04] | | .310 |
|  |  | Social weekend (vs. week) | 0.53 | [.44 – 0.65] | | <.001 | 0.64 | [0.53 – 0.8] | | <.001 |
|  |  | Age | 0.78 | [0.71 – 0.85] | | <.001 | 0.88 | [0.79 – 0.99] | | .033 |
|  |  | Gender | 0.94 | [0.67 – 1.33] | | .744 | 1.44 | [0.64 – 3.22] | | .375 |
|  |  | Race | 0.93 | [0.82 – 1.06] | | .281 | 1.01 | [0.84 – 1.21] | | .938 |
|  |  | Baseline drinking amount | 0.88 | [0.79 – 0.98] | | .024 | --- | --- | | --- |
|  |  | Baseline drinking frequency | 0.66 | [0.43 – 1.03] | | .065 | --- | --- | | --- |
|  | Random effects | | Variance | | SD |  | Variance | | SD |  |
|  | Intercept | |  |  |  |  |  |  |  |  |
|  | Participant ID* Group ID | | <.001 |  | <.001 |  | 1.822 |  | 1.350 |  |
|  | Participant ID | | <.001 |  | <.001 |  | .788 |  | .887 |  |

***Note.*** Study 1: 3443 observations nested within 101 participants across 10 groups; Study 2: 6171 observations nested within 199 participants across 24 groups. Perspective = perspective-taking; Mindful = mindfulness. The zero-inflation sub-model of the hurdle model presented here estimates the probability of an extra zero (no alcohol use) such that a positive estimate indicates a higher chance of no alcohol use. Baseline drinking measures were only collected in Study 1, but not in Study 2. Participants reported their habitual beer, wine, and spirits consumption per week in the month prior to the intervention using the Alcohol Use Questionnaire (AUQ)[^7^](https://paperpile.com/c/X7ucKN/flLF). To create the baseline drinking measures, we averaged the drinking amount and drinking occasion frequency across beer, wine, and spirits.

**Table S4. Group differences in number of drinks pers occasion: active weeks .vs control group**

|  |  | **Study 1** | |  |  | **Study 2** | |  |  |
| --- | --- | --- | --- | --- | --- | --- | --- | --- | --- |
| Fixed Effects | | OR | 95%CI | | *p* | OR | 95%CI | | *p* |
|  | Intercept | 1.22 | [0.20-7.36] | | .825 | 1.52 | [0.23-9.96] | | .662 |
|  | Mindfulness condition (vs. control) | 0.82 | [0.60-1.10] | | .188 | 1.36 | [0.91-2.02] | | .134 |
|  | Perspective condition (vs. control) | 0.69 | [0.49-1.10] | | .029 | 0.96 | [0.63-1.46] | | .839 |
|  | Signal count | 0.99 | [0.98-1.00] | | .007 | 1.00 | [0.99-1.01] | | .675 |
|  | Number of responses | 0.99 | [0.97-1.00] | | .125 | 1.01 | [0.99-1.03] | | .295 |
|  | Social weekend (vs. week) | 1.46 | [1.19-1.78] | | <.001 | 1.41 | [1.13-1.75] | | .002 |
|  | Age | 1.07 | [1.00-1.14] | | .064 | 0.99 | [0.93-1.05] | | .762 |
|  | Gender | 0.89 | [0.67-1.18] | | .417 | 0.61 | [0.40-0.91] | | .017 |
|  | Race | 0.97 | [0.88-1.07] | | .599 | 1.01 | [0.90-1.13] | | .888 |
|  | Baseline drinking amount | 1.20 | [0.64-1.20] | | <.001 | --- | --- | | --- |
|  | Baseline drinking frequency | 0.88 | [2.70-8.85] | | 0.409 | --- | --- | | --- |
| Random effects | | Variance | | SD |  | Variance | | SD |  |
| Intercept | |  |  |  |  |  |  |  |  |
| Participant ID* Group ID | | .048 |  | .219 |  | .375 |  | .612 |  |
| Participant ID | | .017 |  | .131 |  | <.001 |  | <.001 |  |

***Note.*** Study 1: 3443 observations nested within 101 participants across 10 groups; Study 2: 6171 observations nested within 199 participants across 24 groups. Perspective = perspective-taking; Mindful = mindfulness. Baseline drinking measures were only collected in Study 1; not in Study 2. Participants reported their habitual beer, wine, and spirits consumption per week in the month prior to the intervention using the Alcohol Use Questionnaire (AUQ)[^7^](https://paperpile.com/c/X7ucKN/flLF). To create the baseline drinking measures, we averaged the drinking amount and drinking occasion frequency across beer, wine, and spirits.

**Table S5. Group differences in drinking occasion frequency: intervention .vs control group**

|  |  | **Study 1** | |  |  | **Study 2** | |  |  |
| --- | --- | --- | --- | --- | --- | --- | --- | --- | --- |
| Fixed Effects | | OR | 95%CI | | *p* | OR | 95%CI | | *p* |
|  | Intercept | 478.70 | [40.20 – 5700.69] | | <.001 | 249.40 | [12.76 – 4873.61] | | <.001 |
|  | Mindfulness condition (vs. control) | 1.08 | [.74-1.60] | | .680 | 0.86 | [0.49-1.49] | | .585 |
|  | Perspective condition (vs. control) | 1.05 | [.69-1.60] | | .820 | 0.85 | [0.48-1.51] | | .576 |
|  | Signal count | 1.02 | [1.01-1.02] | | <.001 | 1.00 | [1.00-1.00] | | .983 |
|  | Number of responses | 1.01 | [0.99-1.04] | | .294 | 1.01 | [0.99-1.03] | | .242 |
|  | Social weekend (vs. week) | 0.51 | [0.44-0.61] | | <.001 | 0.57 | [0.49-0.67] | | <.001 |
|  | Age | 0.83 | [0.76-0.91] | | <.001 | 0.87 | [0.78-0.97] | | .015 |
|  | Gender | 0.94 | [0.66-1.32] | | .716 | 1.21 | [0.60-2.46] | | .597 |
|  | Race | 0.89 | [0.78-1.01] | | .077 | 0.97 | [0.82-1.15] | | 0.732 |
|  | Baseline drinking amount | 0.91 | [0.82-1.02] | | .108 | --- | --- | | --- |
|  | Baseline drinking frequency | 0.50 | [0.32-0.80] | | .003 | --- | --- | | --- |
| Random effects | | Variance | | SD |  | Variance | | SD |  |
| Intercept | |  |  |  |  |  |  |  |  |
| Participant ID* Group ID | | <.001 |  | <.001 |  | 1.869 |  | 1.367 |  |
| Participant ID | | <.001 |  | <.001 |  | 0.651 |  | .807 |  |

***Note.*** Study 1: 5140 observations nested within 101 participants across 10 groups; Study 2: 9405 observations nested within 205 participants across 24 groups. Perspective = perspective-taking; Mindful = mindfulness. Baseline drinking measures were only collected in Study 1, but not in Study 2.

**Table S6. Group differences in number of drinks pers occasion: intervention .vs control groups**

|  |  | **Study 1** | |  |  | **Study 2** | |  |  |
| --- | --- | --- | --- | --- | --- | --- | --- | --- | --- |
| Fixed Effects | | OR | 95%CI | | *p* | OR | 95%CI | | *p* |
|  | Intercept | 2.86 | [0.55-14.90] | | .212 | 2.46 | 0.52-11.73 | | .258 |
|  | Mindfulness condition (vs. control) | 0.79 | [0.62-1.02] | | .070 | 1.44 | 1.04-1.99 | | .028 |
|  | Perspective condition (vs. control) | 0.74 | [0.56-.98] | | .038 | 0.80 | 0.56-1.14 | | .104 |
|  | Signal count | 0.99 | [0.99-1.00] | | .008 | 1.00 | 1.00-1.01 | | .214 |
|  | Number of responses | 0.99 | [0.97-1.01] | | .225 | 1.00 | 0.98-1.01 | | .816 |
|  | Social weekend (vs. week) | 1.44 | [1.22-1.69] | | <.001 | 1.40 | 1.18-1.65 | | <.001 |
|  | Age | 1.02 | [0.96-1.09] | | .442 | 0.99 | 0.94-1.04 | | .697 |
|  | Gender | 0.80 | [0.63-1.02] | | .071 | 0.66 | 0.47-0.91 | | .011 |
|  | Race | 0.99 | [0.91-1.08] | | .822 | 1.00 | 0.91-1.09 | | .917 |
|  | Baseline drinking amount | 1.15 | [1.08-1.23] | | <.001 | --- | --- | | --- |
|  | Baseline drinking frequency | 0.89 | [2.95-8.01] | | 0.415 | --- | --- | | --- |
| Random effects | | Variance | | SD |  | Variance | | SD |  |
| Intercept | |  |  |  |  |  |  |  |  |
| Participant ID* Group ID | | .064 |  | .253 |  | <.001 |  | .542 |  |
| Participant ID | | .019 |  | .138 |  | <.001 |  | <.001 |  |

***Note.*** Study 1: 5140 observations nested within 101 participants across 10 groups; Study 2: 9405 observations nested within 205 participants across 24 groups. Perspective = perspective-taking; Mindful = mindfulness. Baseline drinking measures were only collected in Study 1, but not in Study 2.

**Supplementary References**

1. [Cosme, D. *et al.* Study protocol: Social health impact of Network Effects (SHINE) study. *PsyArXiv* (2022) doi:](http://paperpile.com/b/X7ucKN/g6J0f)[10.31234/osf.io/cj2nx](http://dx.doi.org/10.31234/osf.io/cj2nx)[.](http://paperpile.com/b/X7ucKN/g6J0f)

2. [Kang, Y. *et al.* Purpose in life, neural alcohol cue reactivity and daily alcohol use in social drinkers. *Addiction* (2022) doi:](http://paperpile.com/b/X7ucKN/kc5K2)[10.1111/add.16012](http://dx.doi.org/10.1111/add.16012)[.](http://paperpile.com/b/X7ucKN/kc5K2)

3. [Zhou, D. *et al.* Mindfulness promotes control of brain network dynamics for self-regulation and discontinues the past from the present. *PsyArXiv* (2021) doi:](http://paperpile.com/b/X7ucKN/jpyGr)[10.31234/osf.io/u83my](http://dx.doi.org/10.31234/osf.io/u83my)[.](http://paperpile.com/b/X7ucKN/jpyGr)

4. [McGowan, A. L. *et al.* Controllability of Structural Brain Networks and the Waxing and Waning of Negative Affect in Daily Life. *Biol Psychiatry Glob Open Sci* **2**, 432–439 (2022).](http://paperpile.com/b/X7ucKN/o0AP)

5. [Suzuki, S. *et al.* Regulation of Craving and Negative Emotion in Alcohol Use Disorder. *Biol Psychiatry Cogn Neurosci Neuroimaging* **5**, 239–250 (2020).](http://paperpile.com/b/X7ucKN/w9vdm)

6. [Bates, D., Mächler, M., Bolker, B. & Walker, S. Fitting Linear Mixed-Effects Models Using**lme4**. *Journal of Statistical Software* vol. 67 Preprint at https://doi.org/](http://paperpile.com/b/X7ucKN/CqqIe)[10.18637/jss.v067.i01](http://dx.doi.org/10.18637/jss.v067.i01) [(2015).](http://paperpile.com/b/X7ucKN/CqqIe)

7. [Townshend, J. M. & Duka, T. Patterns of alcohol drinking in a population of young social drinkers: a comparison of questionnaire and diary measures. *Alcohol Alcohol* **37**, (2002).](http://paperpile.com/b/X7ucKN/flLF)
